# A capsidless (+)RNA yadokarivirus hosted by a dsRNA virus is infectious as particles, cDNA, and dsRNA

**DOI:** 10.1128/jvi.02166-24

**Published:** 2025-02-13

**Authors:** Muhammad Fadli, Sakae Hisano, Guy Novoa, José R. Castón, Hideki Kondo, Nobuhiro Suzuki

**Affiliations:** 1 Agrivirology Laboratory, Institute of Plant Science and Resources, Okayama University 53357 , Kurashiki, Okayama, Japan; 2 Department of Structure of Macromolecules, Centro Nacional Biotecnología (CNB-CSIC), Campus de Cantoblanco, Madrid, Spain; University of Michigan Medical School, Ann Arbor, Michigan, USA

**Keywords:** yadokarivirus, hetero-encapsidation, partner dsRNA virus, fungal virus, *Aspergillus foetidus*, neo-lifestyle

## Abstract

Capsidless yadokariviruses (members of the order *Yadokarivirales*) with (+)RNA genomes divert the capsid of their partner icosahedral double-stranded RNA (dsRNA) viruses in different families of the order *Ghabrivirales* into the replication site. A yadokarivirus, AfSV2, has been reported from a German strain of the ascomycete fungus *Aspergillus foetidus* coinfected by two dsRNA viruses, a victorivirus (AfSV1*,* family *Pseudototiviridae*) and an alternavirus (AfFV, family *Alternaviridae*). Here, we identified AfSV1 as the partner of AfSV2 in a Japanese *A. foetidus* strain after showing the infectiousness of AfSV2 in three forms: virus particles (heterocapsid), transforming full-length complementary DNA (cDNA), and purified replicated form (RF) dsRNA that is believed to be inactive as a translational template. Virion transfection of virus-free *A. foetidus* protoplasts resulted in the generation of two strains infected either by AfSV1 alone or by both AfSV1 and AfSV2. Transformants with AfSV2 full-length cDNA launched AfSV2 infection only in the presence of AfSV1, but not those with AfSV2 RNA-directed RNA polymerase mutant cDNA. The purified fractions containing AfSV2 RF dsRNA also launched infection when transfected into protoplasts infected by AfSV1. Treatment with dsRNA-specific RNase III, but not with proteinase K, S1 nuclease, or DNase I, abolished the infectivity of AfSV2 RF dsRNA. Furthermore, we confirmed the infectiousness of gel-purified AfSV2 RF dsRNA in the presence of AfSV1. Taken together, our results show the unique infectious entity of AfSV2 and the expansion of yadokarivirus partners in the family *Pseudototiviridae* and provide interesting evolutionary insights.

**IMPORTANCE:**

The viral phylum *Pisuviricota* accommodates members with both double-stranded RNA (dsRNA) and (+)RNA genomes. Some members of the second group display peculiar virus lifestyles. These include (+)RNA yadkariviruses, which are capsidless and highjack the capsid of their partner dsRNA viruses in the order *Ghabrivirales* of a different phylum *Duplornaviricota*. We identified the partner dsRNA virus (AfSV1, a victorivirus) of a yadokarivirus (AfSV2) from the ascomycete *Aspergillus foetidus*. AfSV2 is infectious in the presence of AfSV1 in three forms: purified particles, transforming full-length complementary DNA, and, surprisingly, the purified replicative form dsRNA. These combined results expand yadokarivirus partner viruses to the family *Pseudototiviridae* and provide evidence for AfSV2 as a unique infectious entity as well as interesting evolutionary insights.

## INTRODUCTION

Yadokariviruses, with a positive-sense, single-stranded RNA ([+]RNA) genome and phylogenetic affinity for animal caliciviruses, belong to relatively newly established taxa (family *Yadokariviridae*, order *Yadokarivirales*) within the previously established phylum *Pisuviricota* (1). This phylum accommodates both double-stranded RNA (dsRNA) viruses and (+)RNA viruses. The dsRNA members of the phylum are expected to utilize virus isometric particles as the site of RNA synthesis (2), as are members of the phylum *Duplornaviricota* (3, 4). The second group of *Pisuviricota* includes encapsidated (+)RNA viruses and capsidless ones, the latter of which are exemplified by yadokariviruses (1), hypoviruses (5), and hadakaviruses (family *Hadakaviridae*) (6), which have peculiar lifestyles or replication cycles (7). Capsidless (+)RNA viruses commonly infect filamentous fungi, and their replicative dsRNA accumulates well in infected cells. Hadakavirids and hypovirids can complete their replication by themselves as independent viral entities, whereas yadokariviruses rely on particular dsRNA viruses for hetero-encapsidation and replication (viability). It is hypothesized that yadokarivirids highjack the capsid of their partner dsRNA viruses and divert it to the replication site, behaving like authentic dsRNA viruses (8–11). Of note, yadokariviruses utilize their own RNA-directed RNA polymerase (RdRP) for replication (8, 11), which distinguishes them from satellite RNA viruses that lack RdRP genes. There seems to be a very high specificity between a yadokarivirid and a partner dsRNA virus. Indeed, swapping is only possible between mutual strains of a yadokarivirus species and strains of a partner dsRNA virus (12). However, a variety of dsRNA viruses have been identified as partners of yadokariviruses from two suborders, *Betatotivirinae* (families *Yadonushiviridae* and *Megatotiviridae*) and *Alphatotivirinae* (families *Megabirnaviridae* and *Botybirnaviridae*), in the order *Ghabrivirales* of the phylum *Duplornaviricota* (7, 12, 13).

A number of the genomic sequences of yadokariviruses have been reported from various filamentous ascomycetous fungi, particularly phytopathogenic ones. However, only a few of them have been characterized biologically and molecularly (12, 13). Researchers have reported yadokariviruses 1 to 4 (YkV1 to YkV4) in a soil-borne phytopathogenic ascomycete, *Rosellinia necatrix*, from distant geographical localities (12, 14). This fungus has a broad host range—over 400 species of plants—and is difficult to control (15, 16). These yadokariviruses have a simple genome structure with one or two open reading frames (ORFs), and they are believed to express two protein products: RdRP and another protein of unknown function. One-ORF genomes encode 2A-like peptides that self-cleave the polyprotein (8). They have different impacts on their partner dsRNA viruses, host fungus, and likely its plant hosts (12). For example, YkV1 enhances the replication of its partner yadonushi virus 1 (YnV1) (10), and reduces mycelial growth and likely virulence to plant hosts, whereas YkV4 suppresses the replication and symptom expression of its partner dsRNA virus, Rosellinia necatrix megatotivirus 1 (RnMTV1) (12). As expected from their (+)RNA genome nature and observed with many other (+)RNA viruses (17, 18), transgenic expression of the genomic positive-sense strand of YkV1, YkV3, and YkV4 can result in the establishment of their infections in the presence of their partner dsRNA viruses (8, 10, 12). Other yadokariviruses, whose dsRNA virus partners have yet to be determined, include a yadokarivirus termed Aspergillus foetidus slow virus 2 (AfSV2, genus *Alphayadokarivirus*) (19) from a German isolate (IMI 41871, also known as NRRL 337) of *Aspergillus foetidus*. This fungal strain is coinfected by two dsRNA viruses: a victorivirus (AsSV1) (20) and an alternavirus with a multisegmented genome, Aspergillus foetidus fast virus (AfFV) (21). Both dsRNA viruses belong to the order *Ghabrivirales* (22).

Polymycoviruses have been reported from filamentous fungi and belong to the family *Polymycoviridae* (23); they show close and distant phylogenetic affinity to hadakaviruses (6) and yadokariviruses (24), respectively. However, polymycoviruses are not classified into the phylum *Pisuviricota* and are not assigned to higher taxa. This group of viruses has at least four genomic segments, each encoding RdRP, methyltransferase, proline-alanine-serine-rich protein (PASrp), and a hypothetical protein. Polymycoviruses accumulate a large amount of their genomic dsRNA, which is associated with PASrp to form either a colloidal RNA–protein complex or a filamentous particle structure (25). Polymycoviruses can initiate infection in the form of deproteinized, purified dsRNA when transfected into host fungal protoplasts without supplying viral replication-associated proteins (25, 26). This feature is distinct from all other known RNA viruses regardless of their genome type: negative-sense, single-stranded (–)RNA, (+)RNA, or dsRNA. The infectivity of dsRNA entities challenges the established theory of molecular biology as no dsRNA molecules act as a translational template (27). How polymycovirus dsRNA can initiate infection or replication in the absence of viral proteins remains elusive.

In this study, we report the identification of the dsRNA virus partner of a yadokarivirus (AfSV2) from a Japanese strain of the ascomycete *A. foetidus* (a synonym of *Aspergillus luchuensis* and *Aspergillus niger*) (28), namely AfSV1, a member of the family *Pseudototiviridae*, and show the AfSV2 infectivity as a purified dsRNA fraction, the replicative form dsRNA, and as transforming full-length complementary DNA (cDNA) and heterocapsid. The use of reverse genetics with the infectious AfSV2 cDNA revealed the necessity of AfSV2-encoded functional RdRP for its replication. Our findings show AfSV2’s unique infectious entity and provide interesting evolutionary insights.

## RESULTS

### Detection of a yadokarivirus in Japanese strains of *A. foetidus* with a potential partner dsRNA virus

**Fig 1:**
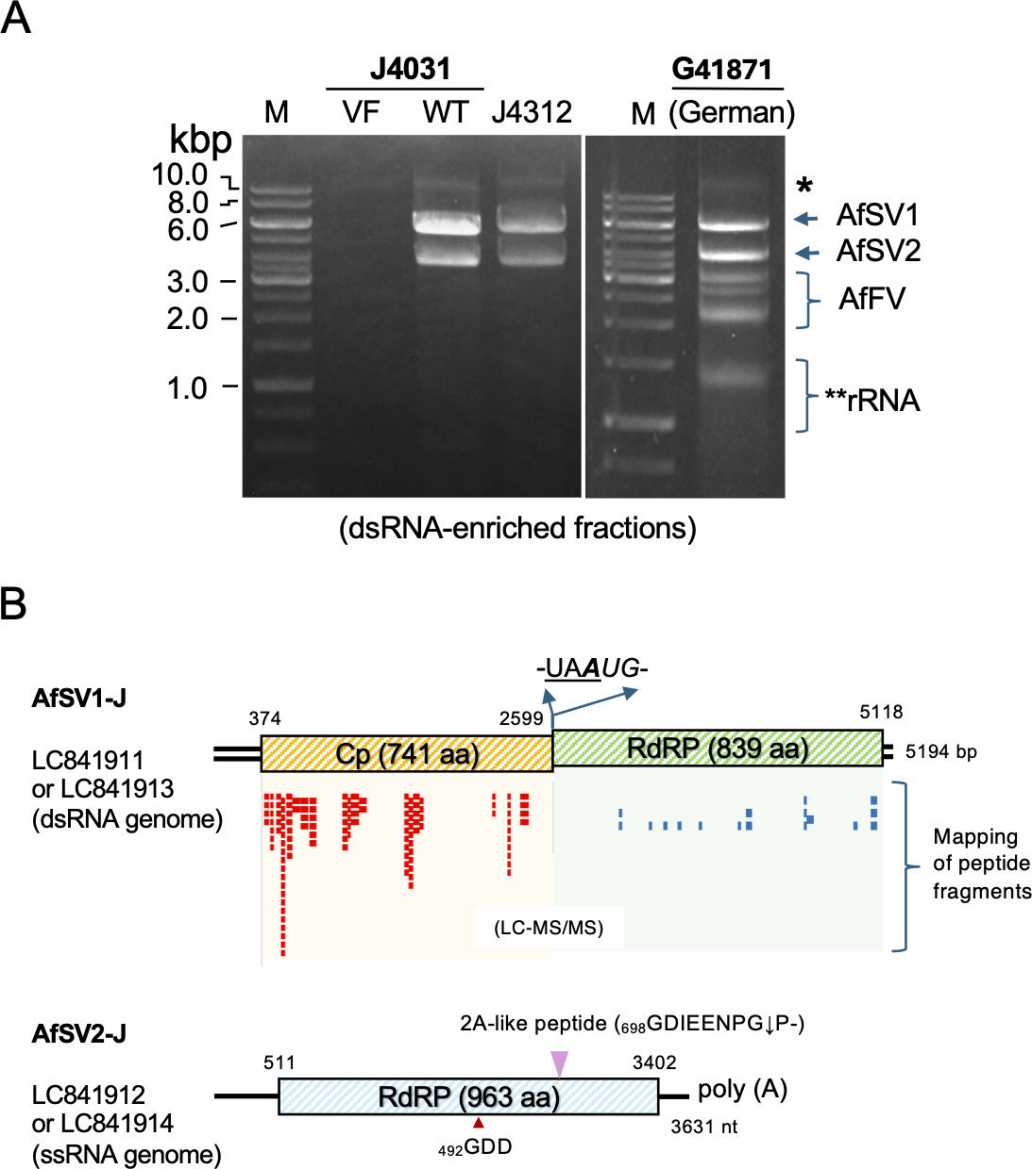
Aspergillus foetidus slow virus 1 and 2 (AfSV1 and AfSV2) co-hosted in two Japanese fungal strains. (**A**) Agarose gel electrophoresis of dsRNA extracted from *A. foetidus* strains J4031 and J4312. dsRNA fractions from the two fungal strains, isolated with cellulose chromatographic purification, were treated with S1 nuclease and DNase I. In parallel, dsRNA fractions from the virus-free strain J4031 VF1 (see Fig. 3 and the associated text) and a German IMI strain (referred to as G41871) were also included. The migration positions of AfSV1 (5.2 kilobase pairs [kbp]) and AfFV (an alternavirus, 3.0–3.6 kbp) genomic dsRNA and the replicative form of AfSV2 dsRNA (3.6 kbp) are shown on the right. The asterisks indicate the band of the undigested genomic DNA or rRNA. The GeneRuler 1 kb DNA ladder (Thermo Fisher Scientific) was used as a size standard in this and subsequent figures. The gel was stained with ethidium bromide. (**B**) Genome organization of AfSV1 and AfSV2 from Japanese *A. foetidus* strain J4031/J4312. AfSV1 is a victorivirus with an undivided dsRNA genome of 5,194 bp. Two ORFs encoding the CP and RdRP are separated by the stop/restart facilitator pentanucleotide, UAAUG. AfSV2 is a yadokarivirus with a non-segmented, positive-sense RNA ([+]RNA) genome of 3,631 nucleotides excluding a 3′ poly(A) tail. The AfSV2 single ORF encodes a putative polyprotein self-cleave 2A-like peptide (GDIEENPG↓P-) located between the RdRP domain and a C-terminal portion of unknown function. The peptide fragments derived from the 75 kDa band in virus particle fraction (see Fig. 2D, lane 12) are shown as small red and light blue bars.

We screened a total of eight strains of *A. foetidus* or closely related *Aspergillus* species (National Institute of Technology and Evaluation [NBRC] 4031, 4118, 4121, 4338, 5708, 4312, 109442, and 109908), most of which likely originated from Japan, for yadokariviruses (Table 1) and candidate dsRNA partner viruses. As a result, we found two strains (NBRC4031 and NBRC4312, hereafter referred to as J4031 and J4312, respectively) (Fig. S1) each with two dsRNA bands with mobilities similar to those of *A. foetidus* slow [S] viruses, AfSV1 and AfSV2 hosted by a German strain (IMI 41871, hereafter referred to as G41871) of *A. foetidus* (Fig. 1A). Sanger sequencing of a cDNA library constructed from strain J4031 revealed that the two dsRNA bands represent the genomic RNA of AfSV1 and the replicative form dsRNA of AfSV2. The virus terminal sequences were determined with Sanger sequencing of rapid amplification of cDNA ends (RACE) clones to obtain the entire genome sequences (accession numbers LC841911 for AfSV1 and LC841912 for AfSV2). Likewise, we also determined the coding complete sequences of AfSV1 and AfSV2 (accession numbers LC841913 and LC841914, respectively) from J4312 based on Sanger sequencing of a cDNA library. The German strain harbored three RNA viruses (*A. foetidus* slow [S] and fast [F] viruses; AfSV1, AfSV2, and AfFV1), as reported earlier (19–21). The viral isolates from J4031 are hereafter referred to as AfSV1-J and AfSV2-J—we used them for further analyses—while those from the German fungal strain are termed AfSV1-G and AfSV2-G. The two Japanese isolates of these viruses are nearly 100% identical in their genomic sequences and share the same genomic organization, with >95% amino acid identity to the corresponding German virus isolates. AfSV1-J possesses two ORFs separated by the tetranucleotide (UAAUG) encoding the capsid protein (CP) and RdRP, respectively, which are likely expressed separately (Fig. 1B). As demonstrated for other mycoviruses (29–31), the AfSV1 RdRP ORF is assumed to be translated via a stop/restart translation mechanism with -UA***A****UG*- (A in bold is shared by the underlined termination and italicized initiation codons). Like the German AfSV2-G isolate, AfSV2-J has a single ORF encoding an RdRP and a 2A-like peptide (GDIEENPG↓P-) (Fig. 1B) that is expected to cleave the preceding RdRP domain, which is believed to be necessary for the virus to replicate (8, 11). The cleaved AfSV2-J RdRP would be 688 amino acids in length. An important difference is at the very 5′ terminal end of the AfSV2 genomic RNA. AfSV2-J had an additional guanine residue (5′-GGGGAUUU-----3′), compared with (5′-GGGAUUU-----3′) identified in the original AfSV2-G in 2013 (19). We determined the entire genomic sequence of AfSV2-G maintained in our laboratory by Sanger sequencing (LC860283). RNA ligase-mediated (RLM)-3′RACE analyses showed that while a majority of AfSV2-G dsRNA molecules appeared to be 5′-GGGAUUU-----3′, a minor population of AfSV2-G contained four guanine residues at the 5′ terminus. In addition, the newly determined AfSV2-G genomic sequence carried several insertions and substitutions, compared to the originally reported AfSV2-G sequence.

**TABLE 1:** *Aspergillus* strains used in this study^*a*^

Fungal strain	Source
*A. foetidus* NBRC 4031 (=J4031)*	Rice malt (Japan?)
*A. foetidus* NBRC 4118	Rice malt (from Nakazawa)
*A. foetidus* NBRC 4121	Unknown (from Nakazawa)
*A. foetidus* NBRC 4312 (=J4312)*	Unknown (from Usami)
*A. foetidus* NBRC 4338	Unknown (from Blochwitz)
*A. foetidus* NBRC 5708	Unknown (from Asai)
*A. awamori* NBRC 109442	Unknown (Japan)
*A. awamori* NBRC 109908	ACTU (Iizula, strain5431)
*A. foetidus* IMI 41871 (=G41871)**	Berlin, Germany

^*a*^ *,**: dsRNA positive fungal strains in bold (harbored viruses: * AfSV1+AfSV2; ** AfSV1+AfSV2+AfFV).

### Identification of AfSV1 as the intrinsic partner dsRNA virus for the yadokarivirid AfSV2

**Fig 2:**
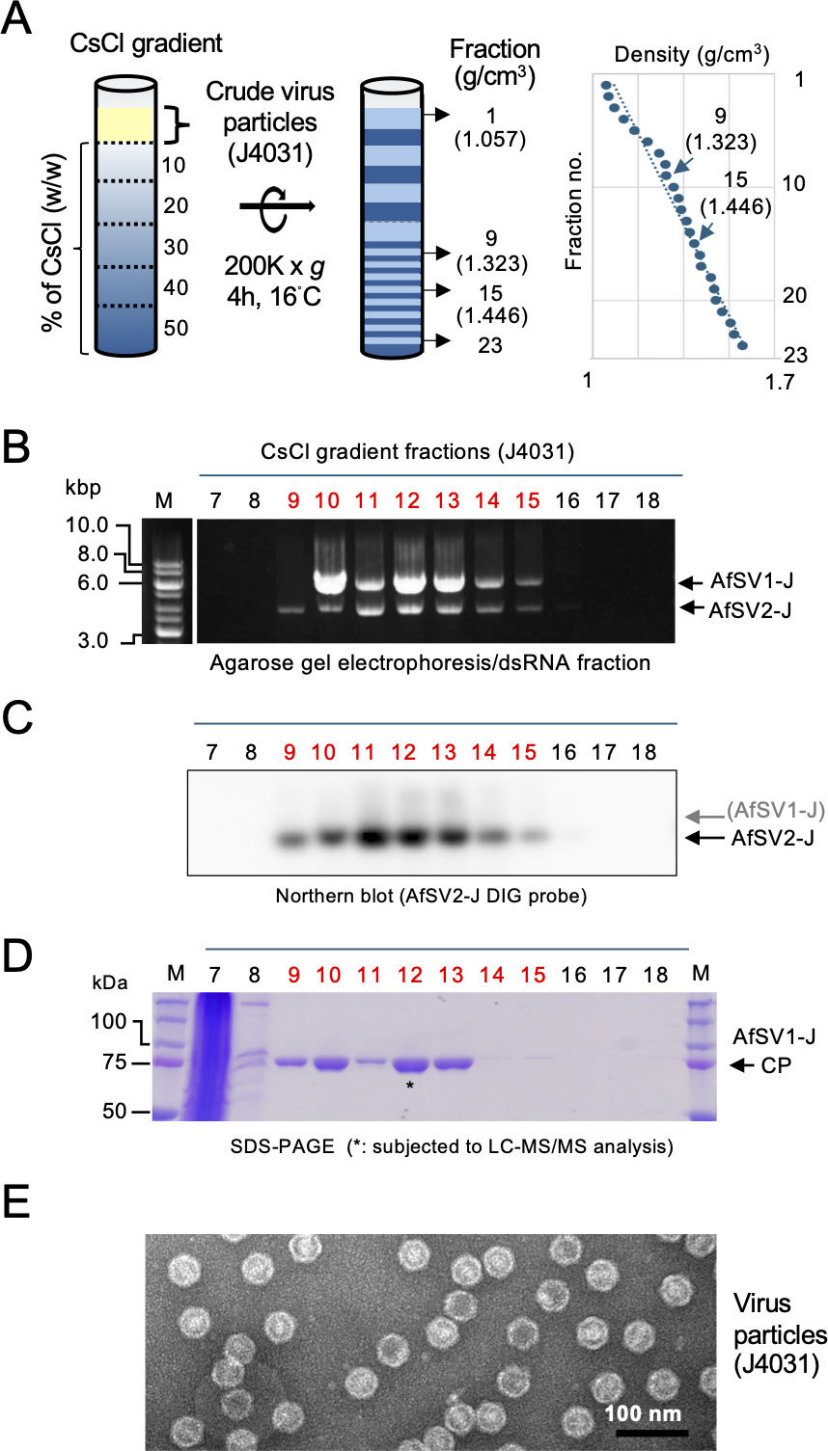
Purification of virus particles from the Japanese *Aspergillus foetidus* strain J4031. (**A**) Cesium chloride (CsCl) gradients before and after ultracentrifugation of crude virus particle preparations from the J4031 strain at approximately 200,000 × *g* for 4 h. The fractions (1–23) and buoyant densities (1.06–1.67 g/m^3^) are shown in the schematic diagram and graph, where the y-axis denotes the density and the x-axis indicates the fraction number. (**B**) The dsRNA agarose gel pattern of fractions obtained after a CsCl equilibrium gradient centrifugation. dsRNA was extracted from each fraction (see Materials and Methods) and electrophoresed in a 1% agarose gel. The panel shows dsRNA samples from fractions of 7–18 (the full gel image is shown in Fig. S2A). Note that fraction 9 (with a density of 1.323 g/cm^3^) likely contained only the replicative form of AfSV2 dsRNA, while fractions 10–15 contained both AfSV1 genomic dsRNA and AfSV2 dsRNA. (**C**) Northern blotting of the replicative form of AfSV2 dsRNA in CsCl equilibrium gradient fractions. The electrophoresed dsRNA preparations from the fractions in panel (**B**) were probed with AfSV2-specific DIG-labeled complementary DNA (cDNA; see Materials and Methods). (**D**) The sodium dodecyl sulfate-polyacrylamide gel electrophoresis (SDS-PAGE) pattern of proteins in the CsCl equilibrium gradient fractions. Protein preparations were obtained from fractions 7–18 as described in Materials and Methods and separated in a 10% polyacrylamide gel. Lane M is a protein size standard (Precision Plus Protein Dual Color Standards, Bio-Rad Laboratories). Proteins were stained with Coomassie Brilliant Blue R250. The protein band in fraction 12 was subjected to liquid chromatography–tandem mass spectrometry. Peptide sequences mapped to the AfSV1-encoded capsid protein are shown in Fig. 1B; Fig. S2B and C. (**E**) Electron microscopy of negatively stained virus particles. The scale bar represents 100 nm. Virus particles were prepared from fungal strain J4031, which harbors both AfSV1 and AfSV2.

Our analyses of the Japanese strain of *A. foetidus* provided a few hints toward the identification of the AfSV2 partner dsRNA virus. First, the two Japanese strains (J4031 and J4312) are coinfected by only two viruses (AfSV1-J and AfSV2-J), unlike the German strain, which is infected by a total of three RNA viruses: AfSV2-G and two dsRNA viruses (AfSV1-G and AfFV) (Fig. 1). This finding ruled out the possibility that AfFV serves as the partner dsRNA virus of AfSV2. Second, we purified virus particles through equilibrium density gradient centrifugation in cesium chloride (CsCl). AfSV1-J genomic dsRNA (5.1 kilobase pairs [kbp]) and AfSV2-J replicative form dsRNA (3.6 kbp) were detectable within a buoyant density range from 1.33 to 1.446 g/cm^3^ (fractions 10–15) when examined with an agarose gel (Fig. 2A and B; Fig. S2A). Based on northern blotting, the band that migrated faster was the AfSV2 replicative form dsRNA (Fig. 2C). Fraction 9 with a buoyant density of 1.323 g/cm^3^ apparently contained only the replicative form of AfSV2-J dsRNA and a single protein band of 75 kDa (Fig. 2B and D). Liquid chromatography–tandem mass spectrometry (LC–MS/MS) confirmed that this protein in fraction 12 is encoded by AfSV1 ORF1, with a coverage of 37% (Fig. 1B; Fig. S2B), strongly suggesting trans-encapsidation of AfSV2-J by AfSV1-J CP. Note that the LC–MS/MS analyses also showed the presence of several peptides corresponding to AfSV1-J RdRP (Fig. 1B; Fig. S2C). Electron microscopy of purified fractions of J4031 that infect AfSV1-J and AfSV2-J revealed isometric particles ~40 nm in diameter (Fig. 2E). No other particle type appeared to be present.

These results strongly suggest that AfSV2 partners with AfSV1 and utilizes the spherical AfSV1 capsid to package AfSV2 RNA.

### Establishment of virus-free strains as well as single and double infectants by virion transfection of the Japanese fungal strain

**Fig 3:**
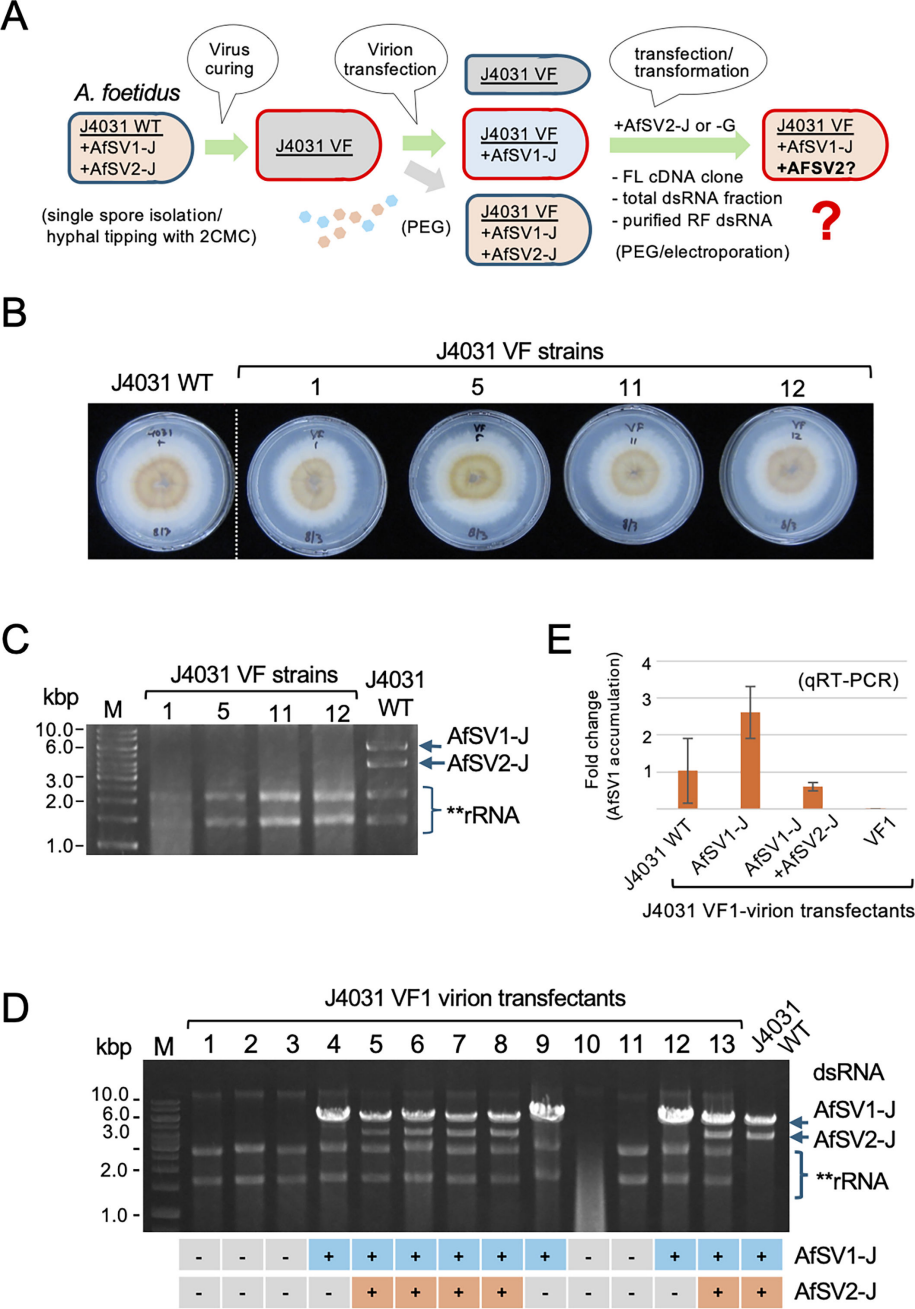
Virus elimination and transfection of *Aspergillus foetidus* J4031. (**A**) The experimental outline of the procedure (see Materials and Methods for additional details). (**B**) Four independent *A. foetidus* virus-free strains (named J4031 VF1, VF5, VF11, and VF12) from the original J4031 strain coinfected by AfSV1 and AfSV2 (J4031 WT). The AfSV1 and AfSV2-free strains were obtained by hyphal tipping with the aid of a nucleotide analog, 2′-C-methyl-cytidine (2-CMC; see Table S1). These strains were cultured on potato dextrose agar (PDA) for 7 days and photographed. (**C**) The absence of virus in these strains was confirmed by agarose gel electrophoresis of dsRNA fractions and also RT-PCR analysis (see the results in Fig. S3). (**D**) Transfection of the virus-free J4031 VF1 strain with virus particles. The virus particles purified from J4031 WT (harboring AfSV1 and AfSV2) were experimentally introduced into protoplasts prepared from J4031 VF1. Randomly selected regenerated isolates were tested for virus infection with a dsRNA assay, in which dsRNA fractions were electrophoresed in a 1% agarose gel. The detection profiles of AfSV1-J and AfSV2-J in the respective colonies are shown at the bottom of the agarose gel. “+” and “–” denote the presence or absence of each virus, respectively. (**E**) RT-qPCR assay of the RNA preparations is shown in Fig. S5. See Materials and Methods for the methodology. The bars indicate the fold change in the AfSV1-J RNA accumulation level normalized to the host actin homolog (centractin ortholog) gene expression. J4031 WT and J4031 VF1 were used as a positive and negative control, respectively.

To transfect different forms of AfSV2, we first attempted to isolate a virus-free strain derived from J4031 using several methods (Fig. 3A). As summarized in Table S1, some commonly utilized methods—single spore isolation, hyphal tipping, heat treatment, and polyethylene glycol (PEG) treatment—did not yield virus-free isolates, even though we examined a large number of subcultures (>28) for each treatment. Thus, we took a combined approach with single spore isolation and hyphal tipping using media supplemented with antiviral and/or antibiotic drugs listed in Table S1. Only attempts with the use of nucleotide analog 2′-C-methyl-cytidine (2-CMC) (32) were successful and yielded four independent virus-free subcultures, termed J4031 VF1, VF5, VF11, and VF12. These VF strains were phenotypically indistinguishable from each other and from the original J4031 strain (Fig. 3B) and tested negative for either virus (AfSV1 or AfSV2) based on a dsRNA assay (Fig. 3C) and reverse transcription PCR (RT-PCR) (Fig. S3). We conducted virion transfection of J4031 VF1 to confirm the infectivity of the purified virions containing AfSV2 heterocapsids and to obtain single and double infectants using virus fractions containing both dsRNAs (Fig. 3A). As with other yadokarivirus–dsRNA virus partner combinations (10), we obtained two types of transfectants in addition to virus-free cultures: one carrying AfSV1-J alone and the other carrying both AfSV1-J and AfSV2-J (Fig. 3D and E). The frequency of each transfectant type is summarized in Table 2; 37.5% (15/40) of the transfectants were doubly infected by AfSV1 and AfSV2, while 17.5% (7/40) were singly infected by AfSV1. We did not obtain a transfectant that was only infected by AfSV2-J, suggesting that AfSV2-J necessitates AfSV1-J for its replication, as expected based on the previous studies with other yadokariviruses (10, 12). Interestingly, the AfSV1-J dsRNA to AfSV2-J dsRNA ratio was apparently retained in the newly established doubly transfected strains, similar to the original J4031 strain, with greater accumulation of AfSV1-J dsRNA in the purified virus fractions and mycelia (Fig. 1A and 3D, lanes labeled J4031 WT). This result is likely different from the case of transfection by multiparticulate dsRNA viruses (partitiviruses), where the ratio of dsRNA1 and dsRNA2 varies in different transfectants (33).

**TABLE 2:** Summary of *A. foetidus* protoplast transfection with virus particle preparations

	AfSV1-J+AfSV2-J	AfSV2-J	Total (positive/tested)		
Experiment I^*a*^	5	3	8/13	2	22/40 (55%)*
Experiment II	10	4	14/27	10	

^*a*^ See Fig. 3D. *, sum values of Experiment I and II.

These data clearly indicate that AfSV1 is a full-fledged dsRNA virus like other victoriviruses, whereas AfSV2 relies on AfSV1 for its replication.

### AfSV2 has a negative impact on AfSV1 accumulation but no effect on host fungal phenotype

It should be noted that all three AfSV1-infected *A. foetidus* strains (J4031, J4312, and G41871) are naturally coinfected by AfSV2. This raises the question of whether coinfection by AfSV2 has a beneficial effect on AfSV1 and/or its host fungus. To answer this question, we biologically compared the three strains and isogenic virus-free strains with J4031. We phenotypically compared the isogenic strains with the J4031 genetic background infected by AfSV1-J and AfSV1-J+AfSV2-J as well as virus-free strains (Fig. S4A, left panel). First, we confirmed the integrity of the virus infections by agarose gel electrophoresis of two RNA fractions: enriched dsRNA and total RNA. We observed the presence of zero, one, or two dsRNA bands of viral origin in the nucleic acid fractions obtained from each of three fungal colonies for VF, AfSV1-J-infected, AfSV1+AfSV2-infected fungi (Fig. 3D). When cultured on potato dextrose agar (PDA), the colony morphology of these strains was indistinguishable regarding the growth rate and pigment production irrespective of virus infection (Fig. S4A, right panel). We also tested whether stress conditions affected their phenotypes. Specifically, we exposed fungal colonies to two different stresses, 1 M sorbitol and 0.05% sodium dodecyl sulfate (SDS), on Czapek’s agar (CZA). Again, there was no discernible difference between uninfected or AfSV1-J- and AfSV1+AfSV2-infected fungal strains, in which the original J4031 strain infected by both viruses was grown in parallel (Fig. S4B).

Agarose gel electrophoretic analyses of the dsRNA-enriched fraction suggested a greater amount of AfSV1-J accumulation in single infections by AfSV1-J compared with double infections by AfSV1-J+AfSV2-J (compare the band intensity of AfSV1-J in Fig. 3D, lanes 4, 9, and 12, with the band intensity in lanes 5–8 and 13). There was a similar pattern for total nucleic acid fractions (Fig. S5, lanes 4–6 vs lanes 1–3 and 7–9). RT-qPCR confirmed that AfSV1-J accumulated approximately twofold to fivefold in double infections compared to single infections (Fig. 3E). Namely, AfSV2-J appeared to interfere with the replication of AfSV1-J.

Taken together, we did not observe the beneficial effects of AfSV2 on AfSV1 or the host fungus. Rather, we conclude that AfSV2 interferes with AfSV1 propagation.

### Full-length AfSV2 cDNA is infectious

**Fig 4:**
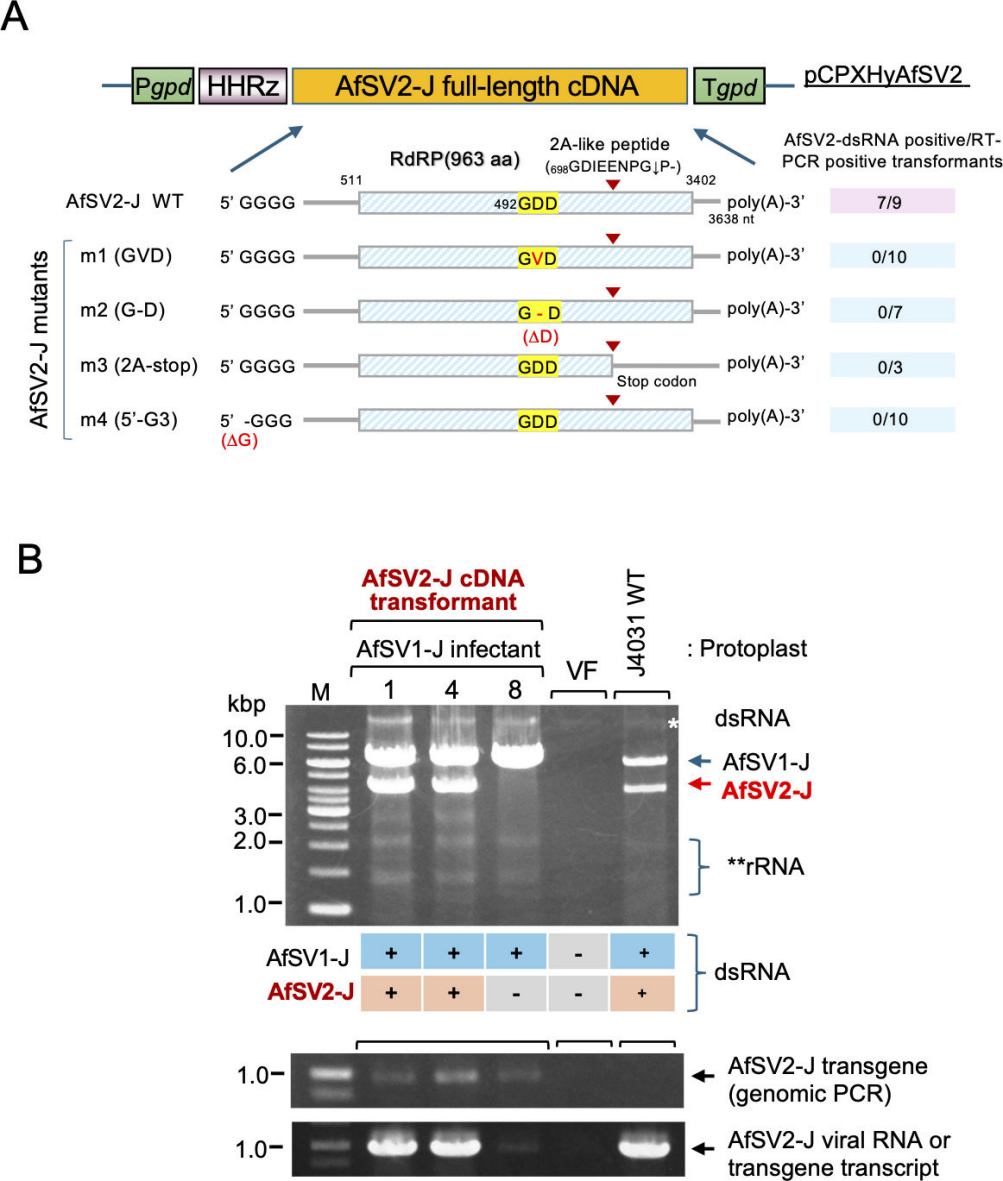
Infectivity of full-length cDNA constructs based on AfSV2-J. (**A**) Full-length cDNA constructs of AfSV2-J in the pCPXHY3 expression vector. Wild-type, full-length AfSV2-J cDNA was inserted into pCPXHY3 (pCPXHyAfSV2-WT) at a site downstream of the hammerhead ribozyme (HHRz) under the control of the *Cryphonectria parasitica* glyceraldehyde-3-phosphate dehydrogenase promoter (P*gpd*) and terminator (T*gpd*), respectively. Likewise, four mutant AfSV2-J cDNA clones (pCPXHyAfSV2m1–pCPXHyAfSV2m4) were prepared. The mutants (pCPXHyAfSV2m1–pCPXHyAfSV2m3) each had a substitution in the RdRP GDD catalytic motif from GDD to GVD, a deletion at the same motif from GDD to GD, or a 2A-like peptide residue to change as a stop codon, which was expected to produce RdRP but no cleaved product at the C-terminal region, whereas pCPXHyAfSV2m4 lacked the 5′-terminal guanine nucleotide sequence. The cDNA clones were used to transform protoplasts derived from the AfSV1-J-infected J4301 strain (Fig. 3). A summary of AfSV2 launching from the wild-type and mutant cDNAs is shown on the right side of each pCPXHY construct. (**B**) Agarose gel analysis of dsRNA-enriched fractions of representative transformants with the wild-type and mutant AfSV1 cDNAs. Extracted dsRNA-enriched fractions were analyzed in 1% agarose gel electrophoresis. The asterisk indicates the genomic DNA band. Colonies were tested for integrated plasmid DNA and AfSV2 cDNA trasncripts (mRNA) or viral RNA (vRNA) expression by one-step colony genomic PCR (gDNA) and RT-PCR (mRNA/vRNA).

For a second AfSV2 inoculum, we inserted full-length cDNA of AfSV2-J into the pCPXHY3 cassette after *Cryphonectria parasitica* glyceraldehyde-3-phosphate dehydrogenase (*gpd*) gene promoter and a 5′ ribozyme sequence (12) (Fig. 4A), and used it to transform *A. foetidus* protoplasts infected by AfSV1-J alone. First, we selected over 100 hygromycin-resistant colonies and subjected them to secondary screening for cDNA integration/transgene transcription by one-step colony RT-PCR. Of nine RT-PCR-positive colonies, all colonies yielded retained AfSV1 as expected, while seven colonies tested positive for AfSV2 (Fig. 4, represented by lanes 1 and 4). These seven colonies harbored the AfSV1 genomic dsRNA and the AfSV2 replicative form dsRNA (Fig. 4B), indicating the infectiousness of the AfSV2-J transcripts derived from the full-length cDNA clone in the presence of a partner dsRNA virus, namely AfSV1-J. The remaining two colonies carrying AfSV2 cDNA but no AfSV2 dsRNA provided only a faint RT-PCR band corresponding to only transgene transcripts (Fig. 4B, lane 8).

Next, we tested four mutant cDNAs: one lacked the 5′-terminal guanine residue (m4) and the others had a deletion or substitutions at the catalytic triad GDD (m2 or m1) or 2A-like peptide (m3) (Fig. 4A). All of these mutations were detrimental to AfSV2-J replication due likely to dysfunction of AfSV2-J cis-factor or RdRP. After transformation as described above, we examined cDNA-integrated colonies. However, none of the PCR-positive colonies were infected by AfSV2-J when assessed with the dsRNA assay (Fig. 4A), suggesting the inability of the mutants to launch infection, is consistent with the previous mutational analyses of other viral RdRPs (34, 35).

These data clearly show that AfSV2 requires its own functional RdRP cleaved by the 2A-like peptide for replication, as is the case for *R. necatrix* yadokariviruses including YkV1 (8, 10, 11).

### The replicative form dsRNA of AfSV2 has an infectious ability

**Fig 5:**
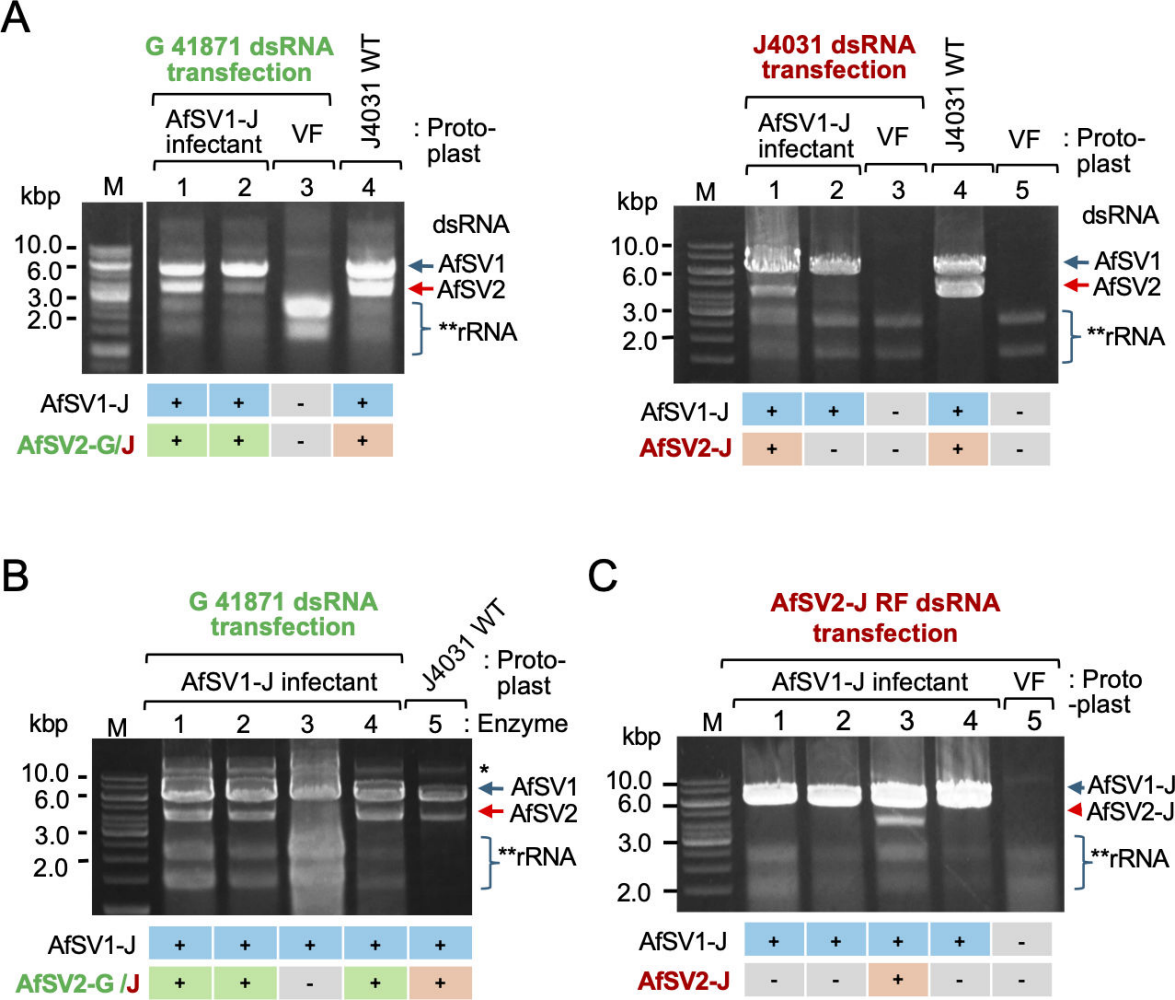
Infectivity of the replicative form of AfSV2 dsRNA to the *Aspergillus foetidus* J4031 strain carrying AfSV1. (**A**) Transfection of AfSV1-J-infected protoplasts with the viral dsRNA fractions. The dsRNA-enriched preparations obtained from the German G41871 strain (infected by AfSV1-G, AfSV2-G, and AfFV) or the Japanese J4031 strain (infected by AfSV1-J and AfSV2-J) were experimentally introduced into the protoplasts. After a dsRNA assay, randomly selected regenerated isolates were tested for AfSV2 infection. The detection profiles of AfSV1-J and AfSV2-G or AfSV2-J in the respective colonies are shown at the bottom of the agarose gel. “+” and “–” denote the presence or absence of each virus, respectively. The J4031 wild-type (WT) dsRNA fraction was loaded as a dsRNA standard. (**B**) The effect of enzymes on the infectivity of viral dsRNA-enriched fractions. dsRNA profiles are shown for representative transfectants with S1 nuclease (lane 1), DNase I (lane 2), RNase III (lane 3), or proteinase K-treated G41871 dsRNA fractions (lane 4). The asterisk indicates the genomic DNA band. See Fig. S6A for treated dsRNA fractions electrophoresed in a 1% agarose gel in 1× TAE buffer and stained with ethidium bromide. (**C**) Infectivity of the replicative form of AfSV2 dsRNA. The purified replicative form of AfSV2-J dsRNA (Fig. S6D) was transfected into AfSV1-J infected protoplasts. Lane 3 shows the dsRNA pattern of an AfSV2-positive transfectant in agarose gel.

As a third type of inoculum, we tested the replicative form of AfSV2 dsRNA for infectivity, an investigation that was prompted by the previous observation that the genomic dsRNA of polymycoviruses, phylogenetically related to yadokariviruses, is infectious (25). For this purpose, we prepared dsRNA-enriched fractions from G41871 infected by AfSV1-G, AfSV2-G, and AfFV (an alternavirus) (Fig. S6A). We used this dsRNA to transfect protoplasts obtained from a Japanese virus-free strain (J4031 VF1) or a fungal strain (originated from J4031) only infected by AfSV1-J (Fig. 3A and D). There were no transfectants with any of the German virus isolates when we used VF fungal protoplasts (Fig. 5A, left panel). Transfection of AfSV1-J-infected protoplasts resulted in the generation of AfSV1-J+AfSV2-G transfectants; out of 27 regenerated colonies, two tested positive for AfSV2-G when examined for dsRNA fractions (Fig. 5A; Table 3). As expected, the remaining AfSV2-G-negative colonies were only infected by AfSV1-J. Note that we did not observe AfFV infection by using RT-PCR when we used dsRNA fractions from the German strain (Fig. S6A). When we used dsRNA-enriched fractions from J4031 (Fig. 1A) for the transfection into AfSV1-J-infected protoplasts, we recovered double infections (AfSV1-J+AfSV2-J) at a rate of 7.4% (4/54; AfSV2-positive/total transfectant tested) (Fig. 5A, right panel).

**TABLE 3:** Summary of AfSV2 dsRNA infectivity

Enzyme treatment or gel purification (lane number)^*a*^		Total transfectants	AfSV2 dsRNA positive	%
Enzyme treatment (G41871 dsRNA fraction)				
(A-1,2)	No treatment	27	2	7.4
(B-1)	S1 nuclease	27	4	11.8
(B-2)	DNase I	27	4	11.8
(B-3)	RNase III	81	0	0
(B-4)	Proteinase K	27	2	7.4
Gel-purified dsRNA (AfSV2-J replicative form dsRNA)				
(C-3)	dsRNA band	27	1	3.7

^*a*^ See representative profiles of dsRNA-positive transfectants; (-number), lanes of panels A (left), B, and C, respectively.

We treated the viral dsRNA-enriched fraction as an inoculum with proteinase K, single-stranded nucleic acid-specific S1 nuclease, dsRNA-specific RNase III, or DNase I, and utilized them for transfection as described above. We aimed to confirm the infectivity of the replicative form of AfSV2 dsRNA and to evaluate the involvement of other factors that may influence the dsRNA infectivity assay. The treated samples showed the expected patterns as viral dsRNAs: only the RNase III-treated sample lost dsRNA bands (Fig. 5B; Fig. S6A), while the proteinase K-treated sample contained no visible protein band except for the one for proteinase K (Fig. S6B). The results of the transfection assay are summarized in Table 3. The transfection of virus-free protoplasts did not allow the accumulation of any viral dsRNAs as in the case of transfection with untreated dsRNA fractions. When we used AfSV1-J-infected protoplasts, we observed AfSV1-J+AfSV2-G double infections at a rate of 7.4%–11.8% for transfections with treated dsRNA fractions (Table 3; Fig. S6C). The only exception was transfection for RNase III treatment, which resulted in loss of infectivity of the fraction: none out of 81 colonies. This strongly suggests that AfSV2-G is infectious as its dsRNA replicative form.

**Fig 6:**
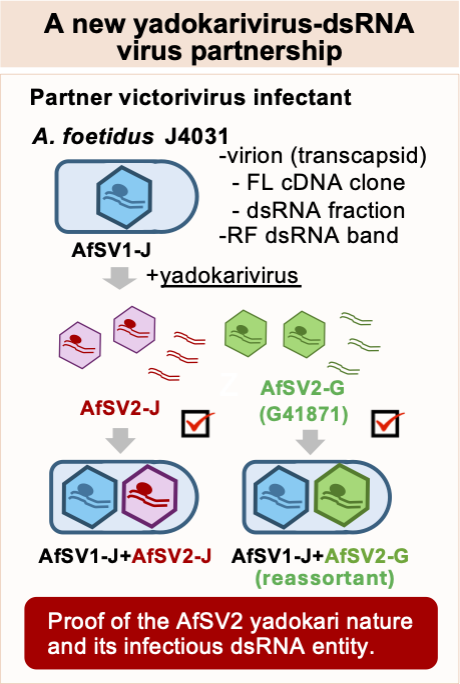
Summary of the proof-of-concept study of the yadokarivirus–dsRNA virus partner interaction in *A. foetidus*. We have demonstrated the infectivity of AfSV2: its virion, cDNA clone, and the replicative form of its dsRNA each represent an infectious entity.

To further confirm the infectivity of the AfSV2-J replicative form dsRNA (3.6 kbp), we purified its dsRNA band from an agarose gel that separated it from the AfSV1-J (5.2 kbp) (Fig. S6D). Then, we used the purified replicative form of AfSV2-J dsRNA to transfect AfSV1-J-infected protoplasts. In this experiment, 3.7% of the transfectants harbored both AfSV1-J and AfSV2-J (Fig. 5C; Table 3). Thus, the J4031 fungal strain was able to host AfSV1-J+AfSV2-J or AfSV1-J+AfSV2-G using the replicative form dsRNA of AfSV2. Importantly, we confirmed the partial sequence of AfSV2-G coinfecting AfSV1-J in the transfectants by Sanger sequencing of RT-PCR fragments , indicating the compatible partnership between AfSV1-J and AfSV2-G (Fig. 6) .

Taken together, our results clearly demonstrated the infectivity of the AfSV2 replicative form dsRNA, but not that of AfSV1 or AfFV. Hence, dsRNA infectiousness is limited to the yadokarivirus AfSV2. We also showed that the Japanese strain of AfSV1 can partner with the German strain of AfSV2, allowing inter-viral partnership between a yadokarivirus and its partner from different fungal strains.

## DISCUSSION

The partnership between capsidless (+)RNA yadokariviruses and dsRNA viruses in the order *Ghabrivirales* represents the yadokari/yadonushi nature, as “yadokari” means “borrowing a house” while “yadonushi” means “landlord” in Japanese (10). To date, the identified dsRNA virus partners of yadokariviruses span several families within the order *Ghabrivirales*, including bi-segmented members of *Botybirnaviridae* (13) and *Megabirnaviridae* (11, 12), and non-segmented members of the families *Yadonushiviridae* (10) and *Megatotiviridae* (12). These virus–virus partnerships have been detected in the fungal classes Leotiomycetes (*Sclerotinia sclerotiorum*) and Sordariomycetes (*R. necatrix*). We identified the partner dsRNA virus of AfSV2 (a yadokarivirus) as AfSV1 (a victorivirus, non-segmented dsRNA virus) and expanded yadokarivirus partners to the family *Pseudototiviridae*. This is the first report of a partnership between the *Alphayadokarivirus* genus and the *Alphatotivirineae* suborder. In addition, we have expanded the host supporting this virus–virus partnership (yadokari/yadonushi nature) to the fungal class Eurotiomycetes (*A. foetidus*).

We demonstrated three infectious forms of AfSV2 (cDNA, heterocapsid, and dsRNA). Nucleic acid-based experimental infectious systems for RNA viruses, whether (+)RNA, (–)RNA, or dsRNA, generally require the transient or transgenic supply of their positive-strand RNA, or its direct introduction into permissive host cells (17, 36–39), because of the need to express virus-encoded, replication-associated proteins. In general, dsRNA is believed to be inactive as a translation template (27), and viral dsRNA is generally non-infectious. This point can be seen in the current study: neither AfFV nor AfSV1 dsRNA could launch infection in several transfection experiments. In addition, long dsRNA is generally absent in eukaryotic cells and thus is readily perceived by a host as a non-self-molecule to elicit robust antiviral defense responses such as RNA silencing (40, 41). Therefore, the observation that the replicative form of AfSV2 dsRNA, whether in a semi-purified or gel-purified form, was able to infect host cells in the presence of AfSV1 as its hetero-capsid-providing partner (Fig. 5 and 6; Fig. S6) was very surprising. As proposed previously, yadokarivirus transcription [synthesis of (+)RNA] and replication [synthesis of (–)RNA] likely occur in the heterocapsid that initially packages its (+)RNA and RdRP. After horizontal and/or vertical transmission of yadokarivirus along with its partner dsRNA virus via anastomosis (hyphal fusion) or sporulation, the hetero-capsid encasing yadokarivirus dsRNA initiates transcription as the first step of the replication cycle. How the replicative form of AfSV2 dsRNA is able to launch infection remains elusive. There may be two possibilities: (i) the introduced dsRNA is packaged in *trans* by AfSV1 and transcribed in the heterocapsid, and (ii) the introduced dsRNA is separated by an as-yet-unidentified host or AfSV1 RNA helicase activities to render the positive strand translationally active. Although the AfSV2 replicative form dsRNA may be incorporated into the heterocapsid, this dsRNA is unlikely to be transcribed in the absence of AfSV2 RdRP. Thus, the second possibility appears to be much more plausible, as active homologous yadokarivirus RdRP has been shown to be essential for the replication of YkV1 (8), YkV3 (11), and AfSV2 (Fig. 4).

Little is known about the mechanism by which a yadokarivirus specifically interacts with its dsRNA virus partner and replicates. Reverse genetics of YkV1 has revealed that (i) YkV1 requires its own RdRP for replication in the presence of its dsRNA partner YnV1 (a member of the family *Yadonushiviridae*) and (ii) the YkV1 2A-like protease is necessary for replication (8, 11). Thus, mutations in YkV1 RdRP and 2A-like catalytic domains abolish YkV1 replication. Based on these observations with YkV1, a model has been proposed to illustrate how yadokarivirus RNA is replicated. The YkV1 transcript is trans-encapsidated by the YnV1 CP, transcribed into dsRNA in the heterocapsid, and then used to synthesize a large amount of YkV1 transcripts that serve as messenger RNA (mRNA) and encapsidation substrates. This model suggests that the replication strategy of YkV1 follows that of genuine dsRNA viruses (4, 42). Our data indicate that this model is applicable to AfSV2, which is hosted by a member of *Pseudototiviridae* (AfSV1), based on the results with reverse genetics (Fig. 4) and hetero-encapsidation (see Fig. 1A and 2D, lane 9). It remains unknown how yadokarivirus RdRP and RNA are specifically packaged by heterologous capsids. Note that the CP of members in different families of the order *Ghabrivirales* share little or no amino acid sequence identity (12, 22), while they commonly make up the *T* = 1 capsid with 60 copies of either CP heterodimers or asymmetric homodimers (43, 44).

The phylum *Pisuviricota* is unique in that it contains both (+)RNA and dsRNA viruses (45, 46). Many of the (+)RNA viruses in the order have peculiar lifestyles, as exemplified by the yadokariviruses. Taking a conventional approach common to (+)RNA viruses (17, 18) and other yadokariviruses within the phylum *Pisuviricota* (8, 10, 11), we established an infectious full-length cDNA clone of AfSV2 (Fig. 4). As mentioned above, we also showed that AfSV2 dsRNA is infectious (Fig. 5 and 6; Fig. S6; Table 3), which is reminiscent of polymycoviruses (classified as dsRNA viruses) with phylogenetic affinity to members of the phylum *Pisuviricota*. Infectivity of the genomic dsRNA of polymycoviruses such as Aspergillus fumigatus tetramycovirus 1 and Colletotrichum camelliae filamentous virus 1 has been demonstrated (25, 26). However, no reverse genetics has been developed for any of the polymycoviruses; thus, the requirement of polymycoviral RdRP for its replication, although likely, or the infectiousness of its transcripts has not yet been substantiated. Members of the family *Polymycoviridae* (23) are phylogenetically more closely related to hadakaviruses with a (+)RNA genome (6) than to yadokariviruses (1). However, hadakavirus dsRNA has been shown to be non-infectious (24), although this may be largely due to technical difficulties resulting from a large number of viral segments (>10 segments). Unlike non-segmented yadokariviruses, members of the other two families, *Polymycoviridae* and *Hadakaviridae*, have multisegmented (<4 segments) RNA genomes. Yadokariviruses have a GDD triad at the RdRP catalytic motif, while hadakaviruses and polymycoviruses have a GDNQ tetrad, which is often found in mononegaviruses with (–)RNA genomes. Members of these three families in the phylum *Pisuviricota* are unable to form rigid particles, although only one polymycovirus has been reported to form filamentous particles (26). What determines the infectivity of the dsRNA and plus-sense transcripts of these related viruses remains unknown. In this regard, the mixed populations of AfSV2-G with different terminal sequences revealed by RLM-3′RACE are noteworthy. A majority of the AfSV2-G negative strand is ---CCC3′ rather than ---CCCC3′ (minor population). We confirmed that the transformed full-length cDNA of the AfSV2-G positive strand, with three guanine residues at the 5′ terminus, is non-infectious (data not shown) as in the case for AfSV2-J. It should be examined in the future whether the positive strand of AfSV2 largely has three or four guanine residues or whether the three G population is infectious in the dsRNA replicative form.

## MATERIALS AND METHODS

### Fungal strains and growth condition

A total of nine strains of *A. foetidus* or closely related species were used in this study. Eight *Aspergillus* spp. strains (NBRC4031, 4118, 4121, 4312, 4338, 5708, 109442, and 109908) largely collected from Japan were purchased from NBRC (Kisarazu, Japan), while one German *A. foetidus* strain (IMI 41871) was obtained from the Centre for Agriculture and Biosciences International (CABI) Europe-UK (Surrey, UK) (Fig. 1A). These fungal strains were cultured on Difco PDA (Becton, Dickinson and Co., Franklin Lakes, NJ, USA), potato dextrose broth (PDB, Becton, Dickinson and Co.), or Czapek’s agar (CZA, Merck, Sigma-Aldrich, St Louis, MO, USA) on the benchtop at 27°C–29°C in the dark unless otherwise mentioned. For long-term storage, mycelial plugs were stored in 10% (vol/vol) glycerol at –80°C. The two Japanese strains, NBRC4031 and NBRC4312, and one German strain, IMI 41871, were most likely molecularly identified as *A. niger* based on a previously described method (28). However, the species names for these strains have been kept as *A. foetidus* in this study to avoid confusion.

For conidia storage, fungi were cultured on PDA, pH 5.0 (adjusted with hydrochloric acid [HCl]) and incubated at 30°C for 10–14 days. The generated spores were collected by scratching the powdery surface with a glass spreader (in 200 µL of sterile water). The suspension was retrieved and filtered through Miracloth (Calbiochem-Merck) into a 50 mL Falcon tube and washed twice with 0.9 M sodium chloride (NaCl) with centrifugation at 4,000 rpm for 15 minutes at 4°C. Finally, the spores were suspended in 50 mL of 0.9 M NaCl and stored at 4°C for up to 1–2 months for direct use in subsequent experiments.

### dsRNA extraction and northern blotting

For dsRNA extraction, fungi were cultured on cellophane-overlayed PDA plates for 3–4 days and harvested by scraping them off from the surface. Mycelia were crushed into a powder in liquid nitrogen and suspended in extraction buffer (100 mM Tris-HCl pH 8.0, 4 mM ethylenediaminetetraacetic acid [EDTA], 100 mM NaCl, and 2% SDS). The suspension was clarified with phenol–chloroform–isoamyl alcohol (PCIA) followed by chloroform–isoamyl alcohol (CIA) (47). The crude extract was incubated with 0.1 g/mL cellulose powder (Advantech, Tokyo, Japan) in STE-ethanol (10 mM Tris-HCl [pH 8.0], 1 mM EDTA, 150 mM NaCl, and 16% ethanol) on a rotary incubator for 1 hour at room temperature. After washing the cellulose three times with STE-ethanol buffer, the cellulose powder with dsRNA was dried, and the dsRNA-enriched fraction was then eluted from the cellulose powder with fresh STE buffer. The elute was concentrated by ethanol precipitation. The dsRNA quality and quantity were checked with 1% agarose gel electrophoresis in 0.5× TAE (40 mM Tris-acetate, 1 mM EDTA, pH 7.8) buffer. The nucleic acids were stained with ethidium bromide.

Northern blotting of dsRNA was performed as described by Sato et al. (11). dsRNA was electrophoresed in 1% (wt/vol) agarose, 1× MOPS (3-[(N-morpholino] propanesulfonic acid), and 1.85% (vol/vol) formaldehyde and probed with cDNA probes labeled with digoxigenin-11-dUTP (DIG) according to the manufacturer’s instruction (Roche, Basel, Switzerland) (11). The primers used for preparing cDNA probes by PCR DIG Labelling Mix (Roche) and for other purposes are listed in Table S2.

### Sequence and phylogenetic analyses of AfSV1 and AfSV2 from Japanese *A. foetidus* strains

For cDNA library construction (48), dsRNA was further purified after DNase and S1 nuclease treatment, followed by PCIA and CIA extraction and ethanol precipitation. Approximately 0.5 µg of dsRNA-enriched fraction (in 50 µL) was mixed with 450 µL of dimethyl sulfoxide (DMSO) and denatured at 65°C for 20 minutes; it was re-collected by ethanol precipitation. cDNAs were synthesized with M-MLV reverse transcriptase (RTase, Invitrogen, Waltham, MA, USA) as described in the manufacturer’s protocol using the random primer (K1, 5′-GCCGGAGCTCTGCAGAATTCNNNNNN-3′). The single-stranded cDNA fragments were used to amplify double-stranded complementary DNAs (dscDNAs) by PCR with the anchor primer (K2, 5′-GCCGGAGCTCTGGAGAATTC-3′) as described by Suzuki et al. (49). The amplified products (dscDNA fragments) were loaded in a 1% agarose gel for size fractionation. A cDNA smear that was >1.5 kbp was cut out from the gel and purified using the Promega Wizard SV Gel and PCR Clean-Up System (Promega, Wisconsin, USA). Purified cDNA fragments were subcloned into pGEM-T Easy Vector (Promega) in *Escherichia coli* DH5α. Colonies with inserts larger than 1 kbp were subjected to Sanger sequencing. A total of 200 colonies were analyzed and assembled using the GENETIX-ATGC ver. 19 software (GENETYX, Tokyo, Japan) to obtain the draft genome sequence.

To determine the terminal sequences, RLM-RACE was performed using the purified dsRNA fractions as described by Suzuki et al. (49). Approximately 20 ng of dsRNA was denatured in 90% DMSO as described above. The denatured dsRNA precipitated with 100% ethanol and 3 M sodium acetate and subjected to 3′-RACE adaptor ligation (5′-PO_4_-CAATACCTTCTGACCATGCAGTGACAGTCAGCATG-3′) by adding T4 RNA ligase (TaKaRa, Kusatsu, Japan) and RNase inhibitor (Toyobo, Osaka, Japan). The ligate was used as a template for cDNA synthesized with M-MLV reverse transcriptase (Promega) in which 3′RACE-1st (5′-CATGCTGACTGTCACTGCAT-3′) complementary to the 3′-half of the 3’RACE-adaptor served as a primer. cDNA was amplified by PCR with a gene-specific primer and 3′RACE-2nd (5′-TGCATGGTCAGAAGGTATTG-3′). After cloning into pGEM-T Easy Vector (Promega), RACE clones were submitted to Sanger sequenced using a BigDye Terminator v3.1 Cycle Sequencing Kit (Thermo Fisher Scientific Inc., Waltham, MA).

Blast searches were run on the protein databases from NCBI (nucleotide or protein collection) (http://blast.ncbi.nlm.nih.gov/Blast.cgi). The phylogenetic relationships of AfSV1 (a victorivirus) and AfSV2 (a yadokarivirus) were analyzed separately based on the deduced amino acid sequence of their RdRPs.

### Purification of virus particles and analysis of dsRNA and protein components

Two hundred microliters of J4031 spore suspension were cultured in 150 mL of PDB for 4 days in a shaker incubator (150 rpm) at 28°C. Approximately 5 g of blot-dried mycelia were ground into powder in liquid nitrogen and suspended in 10 × 50 mL of 0.05 M sodium phosphate buffer (PB, pH 7.0). The suspension was centrifuged at 11,000 rpm (Kubota JA 25.50, Tokyo, Japan) for 30 minutes to clarify. The supernatant was centrifuged at 36,000 rpm for 1.5 hours at 4°C to pellet virus particles in an Optima L-100 ultracentrifuge (Beckman Coulter, Inc., Brea, CA, USA). The crude particle preparation was resuspended in 2 mL of 0.05 M PB (pH 7.0) and loaded on the 10%–50% (wt/wt) CsCl equilibrium density gradient. After centrifuging at 36,000 rpm (SW41Ti rotor) for 18 hours at 16°C, fractions were collected milliliter by milliliter from the top using a bore pipette and subsequently analyzed (12). For dsRNA analysis, 50–100 µL of each fraction was treated with the same phenol–chloroform and cellulose purification method. For protein analysis and virion transfection, each fraction was once diluted with two volumes of 0.05 M PB and centrifuged at 36,000 rpm (70.1Ti rotor) for 1.5 hours at 4°C for concentration. Each pellet was resuspended in 20–30 µL of 0.1 M PB and stored at –80°C until use. Sodium dodecyl sulfate-polyacrylamide gel electrophoresis (SDS-PAGE) and peptide mass fingerprinting of a major protein band from the SDS-PAGE gel were conducted as described previously (50).

Samples (approximately 5 µL) were applied to glow-discharged carbon-coated grids for 2 minutes and negatively stained with 2% (wt/vol) aqueous uranyl acetate. Images were recorded with a JEOL JEM-1400 electron microscope (JEOL Ltd., Tokyo, Japan) operated at 120 kV, with a 4K ONEVIEW CMOS (Gatan, Warrendale, PA, USA) at a nominal magnification of 25,000×.

### Full-length *A. foetidus* yadokarivirus cDNA constructs

Full-length cDNA of wild-type *A. foetidus* yadokarivirus (AfSV2) was generated by RT-PCR and sub-cloned in pGEM-T Easy (Promega), and then transferred to pCPXHY3 (31) after confirming its sequence integrity. The cDNA in pCPXHY3 was placed between the hammerhead ribozyme (HHRz) at the 5′-terminus (11), using the In-Fusion HD Cloning Kit (TaKaRa). The plus-sense transcripts are expected to be produced under the *Cryphonectria parasitica gpd* gene promoter and terminator present in pCPXHY3 (31). Several mutations were introduced into the RdRP catalytic triad GDD motif and the extreme 5′ ends, as described by Das et al. (8) and cloned into pCPXHY3. These constructs were used, after confirming their sequence integrity by Sanger sequencing, to transform protoplasts of an AfSV1-infectant of the virus-cured *A. foetidus* strain J4031(see below).

### Transformation, transfection, and protoplast fusion

To prepare a spore suspension, the fungus was cultured on PDA pH 5.0 (adjusted with HCl) and incubated at 30°C in semi-dark conditions for 10–14 days to induce sporulation. The spores were collected by dropping 200 µL of sterile Milli-Q water onto the powdery surface and scratching the surface with a spreader. The suspension was removed and filtered through Miracloth (Calbiochem, La Jolla, CA, USA) into a 50 mL Falcon tube. The spores were washed twice with 0.9 M NaCl (centrifugation at 4,000 rpm in Kubota JA 25.50 for 15 minutes at 4°C). Finally, the spores were suspended in 50 mL of 0.9 M NaCl—an approximate concentration of 1 × 10^5^ spores/mL—and stored at 4°C for up to 1–2 months. The spore suspension from NBRC4031 was inoculated and germinated in 20 mL of PDB. In less than 24 hours, the sprout mycelia were filtered by Miracloth (Calbiochem) and washed with 0.6 M magnesium sulfate (MgSO_4_). Approximately 20 g of blot-dried mycelia were suspended in 50 mL of the cell wall-digestion solution containing 0.333 g of lysing enzyme (Merck, Sigma-Aldrich, St Louis, MO, USA), 500 µL of beta-glucuronidase (Merck, Sigma-Aldrich), and 0.5 g of bovine serum albumin in an osmotic medium (1.2 M MgSO_4_ in 10 mM NaH_2_PO_4_, pH 5.8). The mixture was incubated for 3–4 hours at 27°C with gentle shaking (70 rpm). The protoplasts were collected from the interface between the digestion mixture and the overlayed trapping buffer (1 M sorbitol and 1 M Tris-HCl, pH 7.0) after centrifugation for 10 minutes at 3,500 rpm, and stored at –80°C until use.

A PEG-mediated method was used for plasmid transformation of the AfSV1-infected J4031 strain (see below) and virus particle transfection of the virus-free J4031 strain, as described by Kanematsu et al. (51), with a slight modification. Approximately, 1 × 10^7^ protoplasts were used for each transformation or transfection experiment, in which plasmid DNA (7–15 µg) or purified particles (approximately 30 µg) were mixed. One week after plating in the regeneration medium, the resulting colonies were transferred to new PDA plates with (for plasmid transformation) or without (for transfection) hygromycin B (80 µg/mL). PCR-based genotyping of fungal colonies using PrimeSTAR GXL DNA Polymerase (TaKaRa) (12) and screening by direct one-step RT-PCR (One Step PrimeScript RT-PCR, TaKaRa) (24, 52) were conducted. In general, 10–20 regenerated colonies were selected for each experiment. The RT-PCR and genomic PCR products were analyzed in a 1% (wt/vol) agarose gel in 0.5× TAE.

### Treatments of dsRNA fractions with nucleases and proteinase

dsRNA fractions obtained as described above were treated aliquoted into five tubes, each containing 10 µg of nucleic acids in a 50 µL of solution, and then treated with an enzyme. The tubes containing DNase I (5 units) (Promega, cat no. M6101) or S1 nuclease (5 units) (Thermo Fisher, cat no. EN0321) were incubated for 30 minutes at 37°C, while the tubes with proteinase K (2.5 µL) (TaKaRa, cat no. 9034) or RNase III (1.5 unit) (cat no. M0245, NEB, Ipswich, MA, USA) were incubated for 1 hour at 37°C. The treated dsRNA was purified with phenol-chloroform and precipitated with 3 M sodium acetate and 100% ethanol. Then, 0.5–2 µg of purified dsRNA was combined with 100 µL of AfSV1-infected J4031 protoplasts (2 × 10^8^/mL) carrying AfSV1 and subjected to electroporation in a Gene Pulser II (Bio-Rad, Hercules, CA, USA) at 1.5 kV and 200 ohms for low range, 500–600 ohms for high range, and 25 µF for the capacitor at a constant time. The cells were plated in regeneration media and incubated at room temperature for 3–5 days. In parallel, dsRNA fractions before treatments were also used for transfection. The regenerated colonies were screened for AfSV2 infection as described above. The enzyme-treated dsRNA and its potential protein association were checked by 1% agarose gel electrophoresis or SDS-PAGE with Coomassie brilliant blue (CBB, G-250) or silver nitrate staining (Invitrogen SilverQuest Silver Staining Kit).

### Virus curing

Hyphal tipping and single spore isolation with or without various drugs (Table S1) were employed to eliminate AfSV1 and AfSV2 from the Japanese *A. foetidus* strain J4031. For hyphal tipping, spore suspensions (see above) were spread on an *Aspergillus* Minimum Media (AMM) plate (containing 2% agar) and cultured at 28°C for 4–7 days. The tip from a single spore germling was taken using a modified glass needle (53) under a dissection microscope (Nikon 101014, Tokyo, Japan), and then cultured on AMM supplemented with drugs and 0.03 mg/mL 2-CMC and incubated at 28°C for 4–7 days (Table S1). The samples were re-cultured five times under the same conditions before being transferred to PDA. For single spore isolation, spore suspensions were diluted appropriately and spread on AMM supplemented with drugs so that single spore germlings could grow separately. One-step RT-PCR and dsRNA extraction were utilized to confirm the virus elimination for obtained fungal subcultures.

## Data Availability

Genome sequences were deposited to NCBI GenBank under accession numbers LC841911 for AfSV1 and LC841912 for AfSV2.

## SUPPLEMENTAL MATERIAL

Supplemental material jvi.02166-24-s0001.pdf Figures S1 to S6; Tables S1 and S2.

ASM does not own the copyrights to Supplemental Material that may be linked to, or accessed through, an article. The authors have granted ASM a non-exclusive, world-wide license to publish the Supplemental Material files. Please contact the corresponding author directly for reuse.
