# Supplementary material for: A capsidless (+)RNA yadokarivirus hosted by a dsRNA virus is infectious as particles, cDNA, and dsRNA
Source: J Virol. 2025 Feb 13;99(3):e02166-24. doi: 10.1128/jvi.02166-24 (PMC11915832; doi:10.1128/jvi.02166-24)
Supplement: Supplemental material — Figures S1 to S6; Tables S1 and S2. [file jvi.02166-24-s0001.pdf]

A capsidless (+)RNA yadokarivirus hosted by a dsRNA virus is infectious as particles, cDNA, and dsRNA

Muhammad Fadli[1], Sakae Hisano[1], Guy Novoa[2], José R. Castón[2], Hideki Kondo[1], and Nobuhiro Suzuki[1+]

Agrivirology Laboratory, Institute of Plant Science and Resources, Okayama University, Kurashiki, Okayama 710-0046[1)]

Department of Structure of Macromolecules, Centro Nacional Biotecnología (CNB-CSIC), Campus de Cantoblanco, 28049 Madrid, Spain

+ Corresponding Author: Dr. Nobuhiro Suzuki
ORCID ID, http://orcid.org/0000-0003-0097-9856
Agrivirology Laboratory
Institute of Plant Science and Resources
Okayama University
Kurashiki, Okayama 710-0046, Japan
Tel. 81(86) 434-1230
Fax. 81(86) 434-1232
e-mail. nsuzuki@okayama-u.ac.jp

Short title: Unique infectious entity of a capsidless (+)RNA yadokarivirus

Supplemental tables, 2; Supplemental figures, 5.

Email addresses: <muhammadfadli@agr.unand.ac.id> (M. Fadli), <shisano@okayama-u.ac.jp> (S. Hisano), <jrcaston@cnb.csic.es> (José R. Castón), <ga.novoa@cnb.csic.es> (Guy Novoa Benavides), <hkondo@okayama-u.ac.jp> (H. Kondo), nsuzuki@okayama-u.ac.jp (N. Suzuki).

Supplementary figure legends

Fig. S1. Colony morphology of two Japanese NBRC strains (referred as J4031 and J4312) of *Aspergillus foetidus*. Fungal colonies were grown on Czapek's agar (CZA) or potato dextrose agar (PDA) for 7 days at 28 °C.

Fig. S2. Purification of virus particles from the Japanese *Aspergillus foetidus* strain J4031. (A) The double-stranded RNA (dsRNA) agarose gel pattern of fractions obtained after cesium chloride (CsCl) equilibrium gradient centrifugation. dsRNA was extracted from each fraction and electrophoresed in a 1% agarose gel. The gel image of the dsRNA samples from fractions 7–19 is also shown in Fig. 2B. (B and C) A summary of the liquid chromatography–tandem mass spectrometry analysis for AfSV1-J capsid protein (CP, B) and RNA-directed RNA polymerase (RdRP, C), respectively. The peptide fragments derived from the AfSV1-J protein bands are also shown in Fig. 1B.

Fig. S3. Virus elimination and transfection of *Aspergillus foetidus* J4031. Four independent virus-free strains (J4031 VF1, VF5, VF11, and VF12) were obtained after subjecting the original J4031 strain, coinfected by AfSV1 and AfSV2, to hyphal tipping with the aid of a nucleotide analog, 2′-C-methyl-cytidine (2-CMC; see also Table S1). The absence of two viruses in the strains was confirmed with RT-PCR analysis with AfSV1-J- and AfSV2-J-specific primers (Table S2). Wild-type *A. foetidus* J4031 was used as a positive control. Agarose gel electrophoresis also confirmed the absence of viral dsRNA accumulation for these strains (see Fig. 3C).

Fig. S4. Colony morphology of *Aspergillus foetidus* infected by AfSV1 and AfSV2 under stress and non-stress conditions. (A) Colony morphology under non-stress conditions. Virus-free J4031 (J4031 VF) was obtained by growing the originally coinfected J4031 strain on potato dextrose agar (PDA) supplemented with 2′-C-methyl-cytidine (2-CMC; Table S1). J4031 VF was transfected with purified virus particles from J4031 to generate singly and doubly infected strains: J4031/AfSV1-J and J4031/AfSV1-J+AfSV2-J (Fig. 3D). These fungal strains were grown on Czapek's agar (CZA, left side panels) or PDA (right side panel) for 7 days at 28 °C. (B) Colony morphology under stress conditions. The fungal strains were cultured on CZA containing 1 M sorbitol or 0.05% sodium dodecyl sulfate (SDS) as a stressor for 7 days at 28 °C.

Fig. S5. Effects of AfSV2 coinfection on AfSV1 accumulation. The agarose gel electrophoresis pattern of total RNA fractions from singly or doubly infected fungal colonies. AfSV1-J genomic double-stranded

RNA (dsRNA) was compared among three subcultures of transfectants of *Aspergillus foetidus* J4031 VF with AfSV1-J or AfSV1-J +AfSV2-J. A total RNA fraction of the original strain J4031 was included as a control. (B) The RT-qPCR assay results of the above RNA preparations are included in Fig. 3E.

Fig. S6. Infectivity of the replicative form of AfSV2 dsRNA to the *Aspergills foetidus* J4031 strain carrying AfSV1. (A) The double-stranded RNA (dsRNA)-enriched preparations obtained from the German strain G41871 (infected by AfSV1-G, AfSV2-G, and AfVF) were treated with different enzymes shown on the top of the gel and as described in Materials and methods. The treated dsRNA fractions were electrophoresed in a 1% agarose gel in 1× TAE buffer and stained by ethidium bromide. The enzyme-treated dsRNA fractions were also used for the transfection of AfSV1-J-infected protoplasts. (B) Protein analysis of G41871 dsRNA-enriched fractions by sodium dodecyl sulfate–polyacrylamide gel electrophoresis. Samples with and without protease K treatment were analyzed in duplicate. (C) Colony phenotypes of the host fungal strains infected by two *A. foetidus* viruses (AfSV1-J+AfSV2-J or AfSV1-J+AfSV2-J) in Czapek's agar (CZA) plates (grown for 7 days at 28 °C). (D) A dsRNA-enriched fraction of J4031 (AfSV1-J and AfSV2-J) and the gel-purified AfSV2 replicative form dsRNA were electrophoresed in a 1% agarose gel in 1× TAE buffer. The dsRNA samples were also used for transfection into AfSV1-J-infected protoplasts. These transfection results are shown in Fig. 5. (E) Sequence comparison of AfSV2 harbored in three fungal infectants by direct Sanger sequencing of RT-PCR fragments. The AfSV2 region at map position 1851-1931 was compared among AfSV2-J in the original J4031 strain, AfSV2-G in the original G41871 strain, and AfSV2-G in a newly transfected J4031 strain carrying AfSV1-J.

Fig. S1

J4312 **J4031**

Aspergillus foetidus

NBRC strains

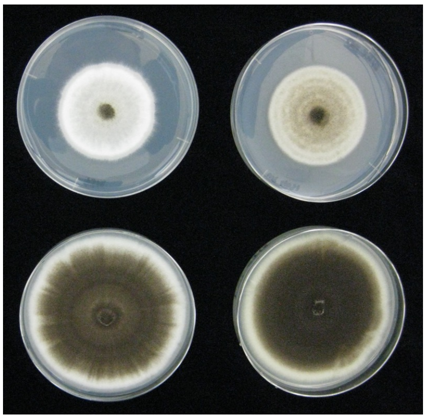

CZA

PDA

(7 days)

Fig. S2

A

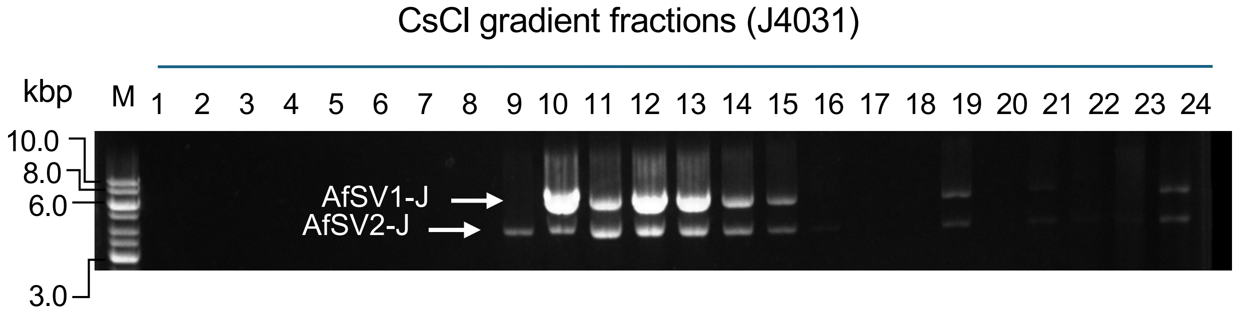

B

Match to: **AfVSL1CP** Score: **2756**

Found in search of 221025_AfVSL1-CP_long.mgf

Nominal mass (M_r): **78556**; Calculated pI value: **5.92**
NCBI BLAST search of AfVSL1CP against nr
Unformatted sequence string for pasting into other applications

Fixed modifications: Carbamidomethyl (C)
Cleavage by Trypsin: cuts C-term side of KR unless next residue is P
Sequence Coverage: **37%**

Matched peptides shown in **Bold Red**

1 MSTIVR**NGYL ASVLANPR**GT R**INGDAQFRR** YRANIR**TEAT VGGVNDARHV**
51 **QVSYEVGR**VH R**TKGAALAAA ADNLVRVEAA YPTAATLVED FIGLAK**KYSN
101 **FSASFEYSSL AGVAER**LAKG **LAASSIWDDV TATDLRGNR**V LNISVLGTHD
151 GPVNSLTNAV FIPRLVNTAV TGDVFTVMIN AVAGEGSAVV TDVLELDAAT
201 R**QPIVATVDT DGFSRACVEA LRIIGANMLA ADQGPLFAYA VTRGIHNVLT**
251 **VVGHTDEGGV SR**DWLRASGF APPFGGIHYG LPVYSGLPAL SSTAPADVSA
301 YVDCIALTSA ALVAHSDPGI TYDDRWYPSF YQGTGADIAE VRPGQHQDGT
351 ADMGRR**NWGQ YTSNMPRFLD YYLPGLGKLF GADGSVQLAC TVARTHSGLM**
401 **PISR**HFNYAS IAPFFWIEPT SLIPHDFLGS PAELNGSGSY CTVDVPREKA
451 GFEDITAAGA ADVAYSAYNV LMRNPRSAWF FAHWLNHPLN GLGAISVRQL
501 DPNGIIHPGA CPDHPDVRDR **VEAALPWTSF MWTR**GQSPFP APGELLNLSG
551 TAGFMVKHLT FDDDAIPTPE HLPTAR**EFEG VSVR**ITCGRP MSIANDTSNS
601 PGSTARRART RAAR**ELAAAT AR**ARLFGRPA VAEMPILTTA PVMRTK**VAAP**
651 **PPQEPPQVSG GVSGWSR**AVP AAENGVSDDR NPDGAAVPVV PHYDSQKFPV
701 VARPQAGAVI GGGAAPAAPG APAADEDDNQ PPQPVAIGNG N

C

Match to: **AfVSL1RdRp** Score: **164**

Found in search of 221025_AfVSL1-CP_long.mgf

Nominal mass (M_r): **92684**; Calculated pI value: **9.31**
NCBI BLAST search of AfVSL1RdRp against nr
Unformatted sequence string for pasting into other applications

Fixed modifications: Carbamidomethyl (C)
Cleavage by Trypsin: cuts C-term side of KR unless next residue is P
Sequence Coverage: **14%**

Matched peptides shown in **Bold Red**

1 MDIQTRISEF GRIGTYLFGL INGTDFMDQI NSHGTYVAKL IALRNSMSSL
51 RERHPLLPAA ASLLLLPFPL QVELSIGDVL LLLRSAFNLS YGTTIQAAIS
101 SNAIPEILSD NLAGRARRIV TLVTQRPDFR EKYFPVKKHP GAQTKANVHL
151 AGLVRSAVVL HGRGIIGR**VL LATAGR**LTDD QASSLLIYGS GLLPTFGALG
201 WAIATHMVLY PDDAKALNGA LKALGTNSTP YGCLLVEANT LQGR**GVGAID**
251 **MR**EKARERCD PDFIQKSVVS ANFDELREHV R**AVITTELSG R**DVKMPTLSD
301 FWTSRWAWCV NGSETAK**SDE VLGLDPK**AYK RTHTRSYRRM AAESVRDEPI
351 STWDGTTYAS ASPKLEAGKT R**AIFACDTR**S YFAFSWCLNA VQKAWRNSRV
401 LLDPGIGGHL GMVNRIRHAQ RGGGVNLMLD FDDFNSQHHT VAMQIVFDEL
451 CKYIGAPDWY RHVLVNSFTR **THVSIDGR**WE SVQGTLMSGH R**GTTFINSVL**
501 **NAAYIR**HAVG GPYFDSLLSL HTGDDVYIRA NTLGDCDYIL NRCRDYGCRL
551 NPAKQSVGYY GAEFLRVAIR GERAYGYFAR GVSGFVNGNW TSSDPLSLTE
601 GLSSAIASCR TLINRSGDAS LADLLGPAIR YRRGLTTR**QT IQLLRGTLSL**
651 **EGSPVFNTNY IIR**NLRVEGV DKETVPIDNR WKRYATTDYL TDHLSPVEVE
701 AIHLAKTDPG PLMIASGYRK GLNLDDKGTP SQVRFRPLPP RLATGFASAS
751 QLTARRAEPG CLAK**YPVLSL IAGR**LTDDEL RRLVAMEGGN NTTSDIRKEA
801 FGESPTSK**NI IGFLSYADAA ALSK**VTSSGN IFTSISVRV

Fig. S3

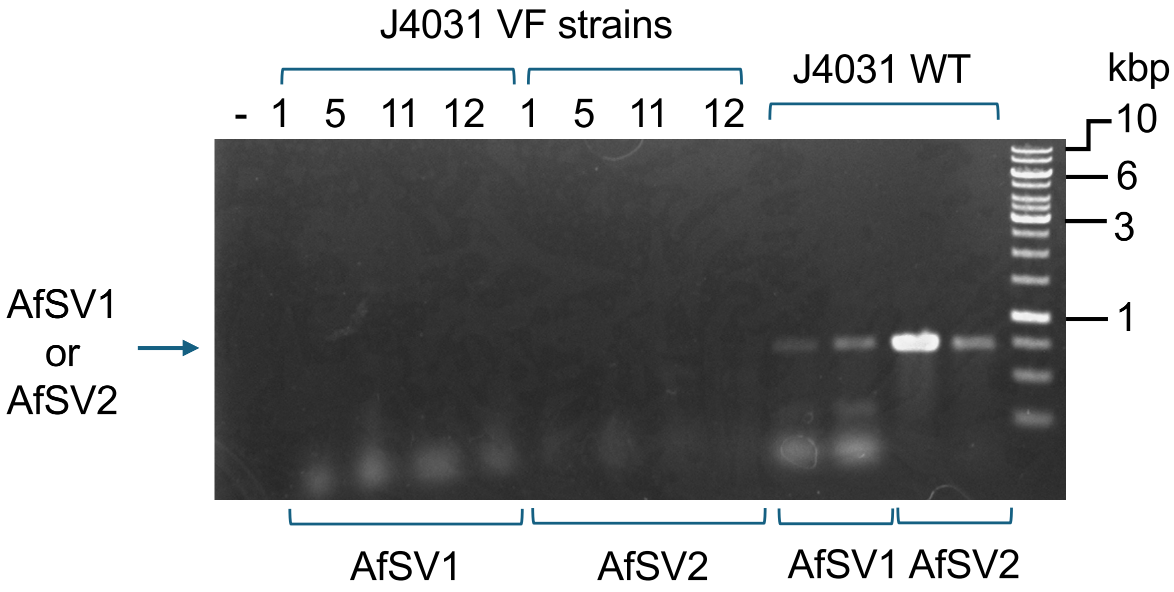
J4031 VF strains
J4031 WT
kbp
- 1 5 11 12 1 5 11 12
10
6
3
1
AfSV1
or
AfSV2
AfSV1
AfSV2
AfSV1 AfSV2

Fig. S4

A

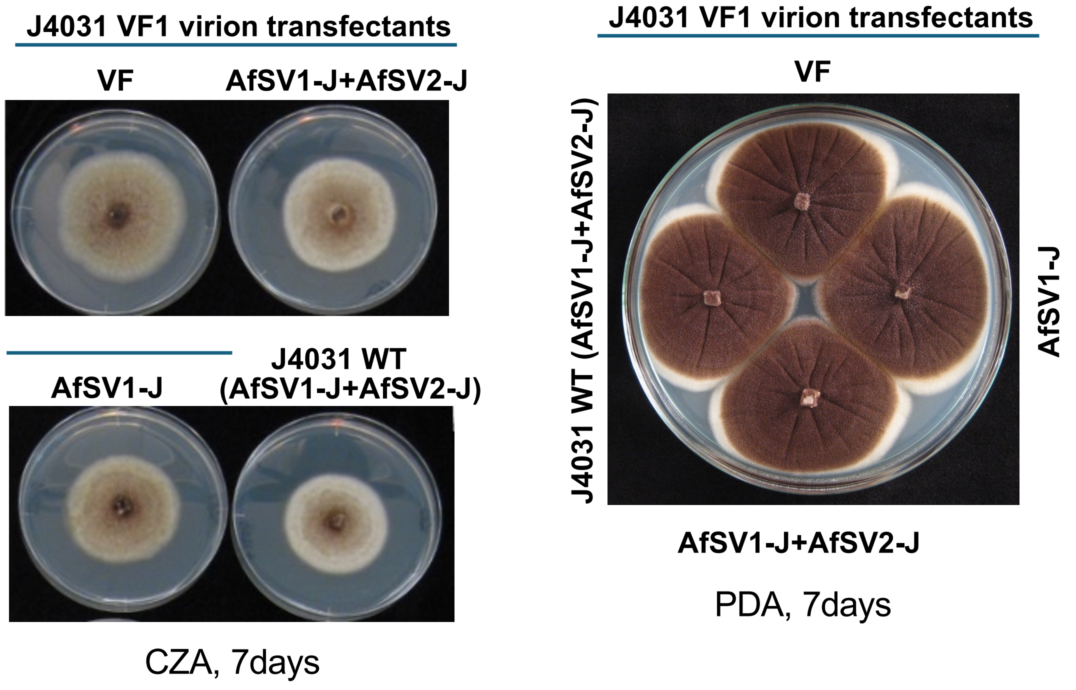
J4031 VF1 virion transfectants
VF
AfSV1-J+AfSV2-J
AfSV1-J
J4031 WT
(AfSV1-J+AfSV2-J)
CZA, 7days
J4031 VF1 virion transfectants
VF
J4031 WT (AfSV1-J+AfSV2-J)
AfSV1-J
AfSV1-J+AfSV2-J
PDA, 7days

B

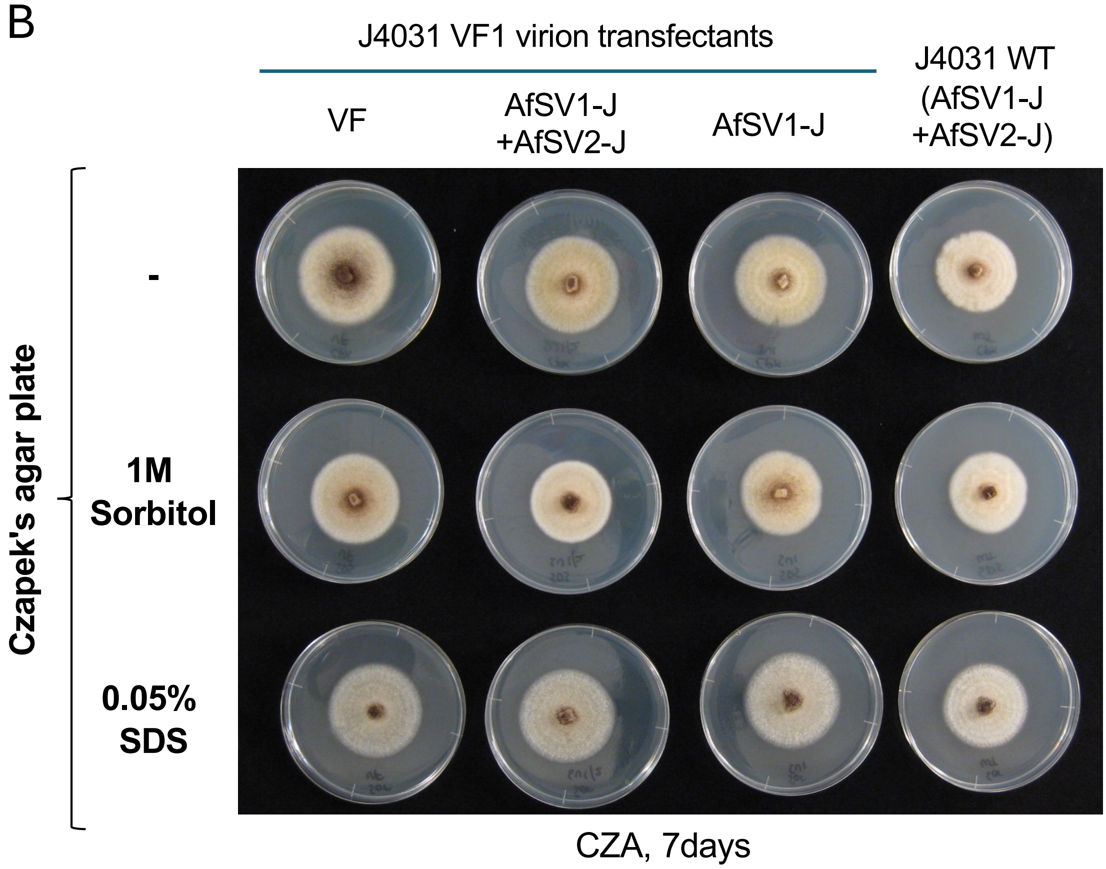
J4031 VF1 virion transfectants
VF
AfSV1-J
+AfSV2-J
AfSV1-J
J4031 WT
(AfSV1-J
+AfSV2-J)
Czapek's agar plate
-
1M
Sorbitol
0.05%
SDS
CZA, 7days

Fig. S5

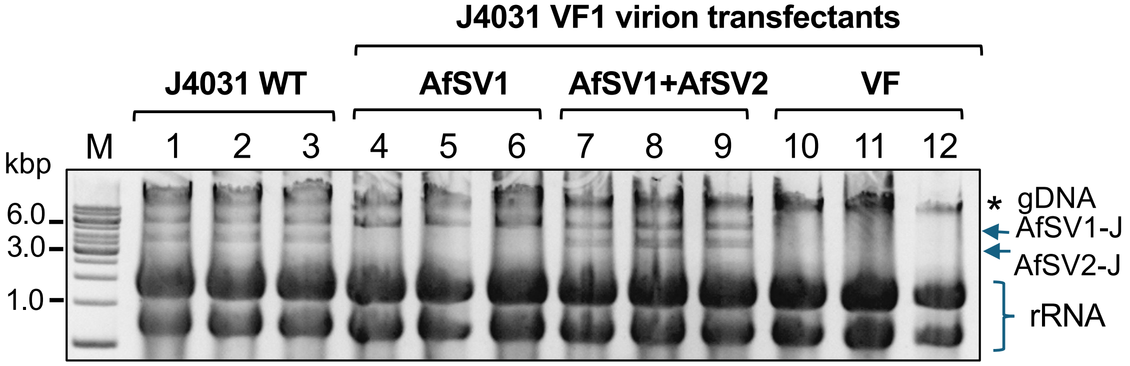

(Agarose gel electrophoresis/total RNA fractions)

Fig. S6

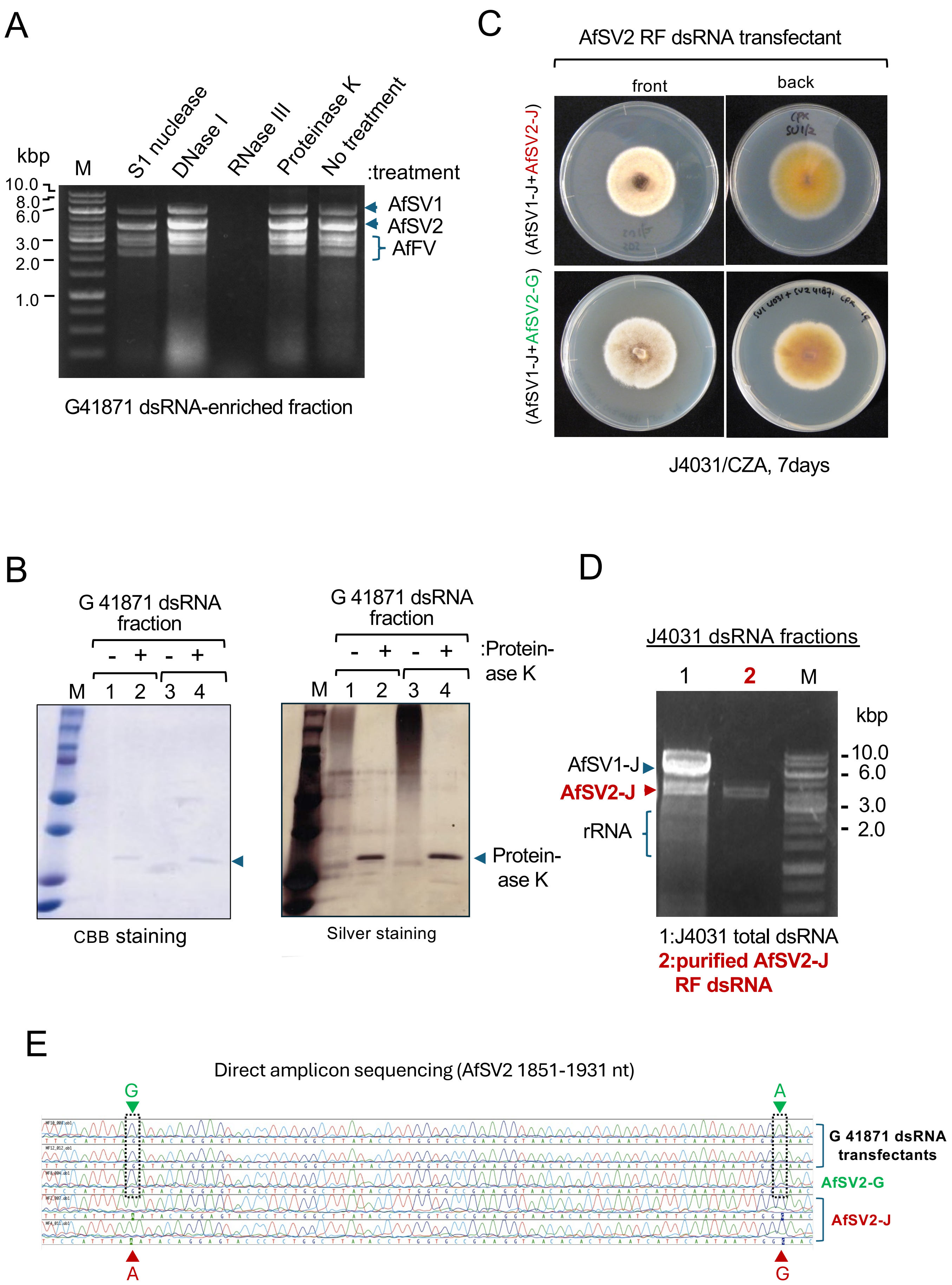
A
kbp
M
S1 nuclease
DNase I
RNase III
Proteinase K
No treatment
:treatment
10.0
8.0
6.0
3.0
2.0
1.0
AfSV1
AfSV2
AfFV
G41871 dsRNA-enriched fraction
C
AfSV2 RF dsRNA transfectant
front
back
(AfSV1-J+AfSV2-J)
(AfSV1-J+AfSV2-G)
J4031/CZA, 7days
B
G 41871 dsRNA fraction
- + - +
M 1 2 3 4
CBB staining
G 41871 dsRNA fraction
- + - +
:Protein-ase K
M 1 2 3 4
Protein-ase K
Silver staining
D
J4031 dsRNA fractions
1 2 M
kbp
10.0
6.0
3.0
2.0
AfSV1-J
AfSV2-J
rRNA
1:J4031 total dsRNA
2:purified AfSV2-J RF dsRNA
E
Direct amplicon sequencing (AfSV2 1851-1931 nt)
G
A
G 41871 dsRNA transfectants
AfSV2-G
AfSV2-J
A
G

Table S1. Virus elimination by various treatments.

No	Methods	Number of sub-isolates tested	Virus free sub-isolates obtained
1	Single spore isolation on:	85	0
	a. 2% water agar (WA)	85	0
	b. Minimum media (MM)*	28	0
	c. MM supplemented with cycloheximide	40	0
2	PEG (polyethylene glycol) matrix in Synthetischer Nährstoffarmer broth	60	0
3	Heat treatment (37ºC, 50ºC and 65ºC in range 3, 6, 12, 24 hours each temperature) of Agar plug on PDA**	53	0
4	Hyphal tipping on:		
	a. MM	50	0
	b. MM supplemented with cycloheximide	53	0
	c. MM supplemented with 2'-C-Methylcytidine (2-CMC)	54	4
	d. MM supplemented with ribavirin	72	0
	e. MM supplemented with 5'-fluorouracil	72	0
	f. MM supplemented with cycloheximide	40	0
5	Repeated subculture on MM supplemented with cycloheximide	40	0

*Czapek's agar (CZA)

** Approximately 12 sub-isolates were tested for each treatment with the different time period.

Table S2. Primer list.

Primers for	Target	Primer sequence (5'-3')	Map position / Reference
RACE-PCR	AfSV1J-5'R	TCAGTAGCCGTCACATCATCC	754<774
RACE-PCR	AfSV1J-3'F	AGGCAGACCATTCAGCTGTTG	4510>4530
RACE-PCR	AfSV2J-5'R	CACCGGGACATAACTTTTCGG	501<521
RACE-PCR	AfSV2J-3'F	GAGATAAGTCCCCATTGCACC	3088>3108
RACE-PCR	RACE adaptor	PO4-CAATACCTTCTGACCATGCAGTGACAGTCAGCATG	Suzuki et al., J Gen Virol (2004) 85: 3437-3448
RACE-PCR	RACE 1st	CATGCTGACTGTCACTGCAT	Suzuki et al., J Gen Virol (2004) 85: 3437-3448
RACE-PCR	RACE 2nd	TGCATGGTCAGAAGGTATTG	Suzuki et al., J Gen Virol (2004) 85: 3437-3448
E. coli colony screening	M13F	GTAAAACGACGGCCAGT	pGEM-T-easy vector system
E. coli colony screening	M13R	GCGGATAACAATTTCACACAGG	pGEM-T-easy vector system
E. coli colony screening	pCPXHy3-Pgpd-F	AAAGAAGGATTACCTCTAAAC	Craven et al., J Virol (1993) 67: 6513-6512
E. coli colony screening	pCPXHy3-Tgpd-R	AACGCCAGCAACGCGGCCTTT	Craven et al., J Virol (1993) 67: 6513-6512
RT-PCR	AfSV2-1F	GGGGATTTTTGMCCGCCCTGA	1>21
RT-PCR	AfSV2-1R	GTAAATTGGTAATTGACGGGG	958<978
RT-PCR	AfSV2-2F	TGCGTCGTATACAAAGAAATA	737>757
RT-PCR	AfSV2-2R	TTTTGCTTGGCGTGTTCCATC	1580<1600
RT-PCR	AfSV2-3F	ACCTATGACCTACAGAGAAGT	1269>1289
RT-PCR	AfSV2-3R	TGGAGAGTACGGTCAGCCTCA	2202<2222
RT-PCR	AfSV2-4F	CGTCAGAATCTCCGGTACCTC	2017>2037
RT-PCR	AfSV2-4R	GAAGACTTGATCCTGGAGGGG	2976<2996
RT-PCR	AfSV2-5F	GAGTTGGCCCGTAATGGCATT	2656>2676
RT-PCR	AfSV2-5R	TTTTTTTCTTGGCTGGGGTTT	3618<3638
RT-PCR	AfSV2_4312-1F	GGGATTTTTGACCGCCCAGAT	1>21
RT-PCR	AfSV2_4312-1R	ATTTAGGATGTTTCGTTATTG	917<937
RT-PCR	AfSV2_4312-2F	TTCGCGAGTCACCTAAAGGTG	701>721
RT-PCR	AfSV2_4312-2R	TTGCTTGGCGTGTTCCATCTA	1578<1598
RT-PCR	AfSV2_4312-3F	CTCAGCAGTTCCTCAAGAATC	1298>1318
RT-PCR	AfSV2_4312-3R	CGGTCAGCCTCAAAGATTGGG	2193<2213
RT-PCR	AfSV2_4312-4F	TGAGATCTTTGAAACCAACAC	1997>2017
RT-PCR	AfSV2_4312-4R	AGGGGTAGGGACAAGGCTCTT	(2960<2981)
RT-PCR	AfSV2_4312-5F	AGAGTTGGCCCGTAATGGCAT	2655>2675
RT-PCR	AfSV2_4312-5R	TTTTTTTCTTGGCTGGGGTTT	3618<3638
RT-PCR	AfSV1-1F	TGAAACGAAAGGCAACTCTGA	1>21
RT-PCR	AfSV1-1R	AAAAACGTCACCGGTCACAGC	(878<898)
RT-PCR	AfSV1-2F	CCCACTGCCGCTACTCTGGTG	617>637
RT-PCR	AfSV1-2R	GCGGCACGTCGACGGTACAAT	1692<1712
RT-PCR	AfSV1-3F	TTCCTGGCCTGGGTAAATTAT	1491>1511
RT-PCR	AfSV1-3R	TATTACGGCTCCAGCTTGGGG	2483<2503
RT-PCR	AfSV1-4F	GTAGCAGAGATGCCGATTCTC	2264>2284
RT-PCR	AfSV1-4R	ATAATCCCTCTCCCATGTAAG	3075<3095
RT-PCR	AfSV1-5F	GCCTTTCCCTCTACAAGTTGA	2796>2816

RT-PCR	AfSV1-5R	GGTTAACCATACCGAGGTGCC	3821>3841
RT-PCR	AfSV1-6F	GCTCTTACCGCCGAATGGCTG	3602>3622
RT-PCR	AfSV1-6R	CGCCCTCAACACGTAGGTTCC	4586>4606
RT-PCR	AfSV1-7F	TAGGGTGGCCATACGTGGCGA	4293>4313
RT-PCR	AfSV1-7R	GCAGTTAGCCCCATACGGGGC	(5188<5194)
RT-qPCR	AfSV1_qRT-F	ATCGACATGCGGGAAAAAGC	3343>3362
RT-qPCR	AfSV1_qRT-R	TCGAAATTGGCCGAGACAAC	3400>3419
RT-qPCR	AfSV2_qRT-F	AACGCCTTTACAAGCACCAC	1625>1644
RT-qPCR	AfSV2_qRT-R	AACGCGGCATTTTGATGTCC	1692<1711
RT-qPCR	Actin F	AGAAGTTCAAGGTGCGCATC	Pel et al., Nat Biotech (2007) 25: 221-231
RT-qPCR	Actin R	ACTTGCGAACAGGAAAACGG	Pel et al., Nat Biotech (2007) 25: 221-231
Northern hybridization	AfSV2_probe-F	AGACGCGATCCCTTTCTCACC	1015>1036
Northern hybridization	AfSV2_probe-R	CTAAGGCGGGCAAGAACACGT	1731<1751
Infectious clone	IF_pCPX-HH-AfSV2-F	TAGAGGTACGCGGCCAAGCTCAAAAATCCCCCTGATGAG<br>TCCGTGAGGACGAAACGGTACCCGGTACCGTCGGGGATT<br>TTTGACCGCCCTGA	Vec(15)+HHrz+(1>21)
Infectious clone	IF_pCPX-AfSV2-R	GTCAAGCATGCGCGGCCTTTTTTTTTTTTTTTTTTTCTTGG<br>CTGGGGTTT	Vec(15)+T20+(3631>3618)
Inoculum amplification	pCPXHy3-12F	AAAGAAGGATTACCTCTAAAC	Craven et al., J Virol (1993) 67: 6513-6512
Inoculum amplification	pCPXHy3-5109R	AACGCCAGCAACGCGGCCTTTTTAC	Craven et al., J Virol (1993) 67: 6513-6512
Mutant clones	AfSV2_GDD>GQD-F	GTTATGGTGAAGATTGGTTGAGA	1979>2002
Mutant clones	AfSV2_GDD>GQD-R	AATCTTCACCATAACGGGAGTCA	1971<1994
Mutant clones	AfSV2_GDD>GVD-F	GTTATGGTGTTGATTGGTTGAGA	1979>2002
Mutant clones	AfSV2_GDD>GVD-R	AATCAACACCATAACGGGAGTCA	1971<1994
Mutant clones	AfSV2_GDD>GAD-F	GTTATGGTGCGGATTGGTTGAGA	1979>2002
Mutant clones	AfSV2_GDD>GAD-R	AATCCGCACCATAACGGGAGTCA	1971<1994
Mutant clones	AfSV2_GDD>GD-F	GTTATGGTGATTGGTTGAGATCT	1979>2005
Mutant clones	AfSV2_GDD>GD-R	AACCAATCACCATAACGGGAGTC	1965<1994
Mutant clones	AfSV2_2A>stop-F	AAAACCCCTGAGGACCCATCAAAT	2594>2615
Mutant clones	AfSV2_2A>stop-R	TGGGTCCTCAGGGGTTTTCTTCTA	2588<2609
Mutant clones	AfSV2_5'G4>G3-F	GGGATTTTTGACCGCCCTGAT	2>23
Mutant clones	AfSV2_5'G4>G3-R	GCGGTCAAAAATCCCGACGGT	(HHrz:6)+(2<16)
Fungal genotyping	ITS1(F)	TCCGTAGGTGAACCTGCGG	Gardes and Bruns, Mol Ecol (1993) 2: 113-118
Fungal genotyping	ITS4(R)	TCCTCCGCTTATTGATATG	Gardes and Bruns, Mol Ecol (1993) 2: 113-118
Fungal genotyping	Calmodulin-F	GCAAGTTTCTGAGTACAAGGA	Hong et al., Appl Microbiol Biotechnol (2014) 98: 555-561
Fungal genotyping	Calmodulin-R	CGAACTCGTTGTCTGGTAGCA	Hong et al., Appl Microbiol Biotechnol (2014) 98: 555-561
Fungal genotyping	B-tubulin-F	TGGTACGTATACAACTGCCAT	Hong et al., Appl Microbiol Biotechnol (2014) 98: 555-561
Fungal genotyping	B-tubulin-R	CCAGTTGTTACCAGCACCGGA	Hong et al., Appl Microbiol Biotechnol (2014) 98: 555-561
